# Contribution of Ion Energy and Flux on High-Aspect Ratio SiO_2_ Etching Characteristics in a Dual-Frequency Capacitively Coupled Ar/C_4_F_8_ Plasma: Individual Ion Energy and Flux Controlled

**DOI:** 10.3390/ma16103820

**Published:** 2023-05-18

**Authors:** Wonnyoung Jeong, Sijun Kim, Youngseok Lee, Chulhee Cho, Inho Seong, Yebin You, Minsu Choi, Jangjae Lee, Youbin Seol, Shinjae You

**Affiliations:** 1 Applied Physics Lab for PLasma Engineering (APPLE), Department of Physics, Chungnam National University, Daejeon 34134, Republic of Korea; 2 Institute of Quantum Systems (IQS), Chungnam National University, Daejeon 34134, Republic of Korea; 3 Samsung Electronics, Hwaseong-si 18448, Republic of Korea

**Keywords:** plasma processing, plasma etching, high-aspect ratio SiO_2_ etching, etching rate, individual plasma internal parameter control, ion flux, ion energy

## Abstract

As the process complexity has been increased to overcome challenges in plasma etching, individual control of internal plasma parameters for process optimization has attracted attention. This study investigated the individual contribution of internal parameters, the ion energy and flux, on high-aspect ratio SiO${}_{2}$ etching characteristics for various trench widths in a dual-frequency capacitively coupled plasma system with Ar/C${}_{4}$F${}_{8}$ gases. We established an individual control window of ion flux and energy by adjusting dual-frequency power sources and measuring the electron density and self-bias voltage. We separately varied the ion flux and energy with the same ratio from the reference condition and found that the increase in ion energy shows higher etching rate enhancement than that in the ion flux with the same increase ratio in a 200 nm pattern width. Based on a volume-averaged plasma model analysis, the weak contribution of the ion flux results from the increase in heavy radicals, which is inevitably accompanied with the increase in the ion flux and forms a fluorocarbon film, preventing etching. At the 60 nm pattern width, the etching stops at the reference condition and it remains despite increasing ion energy, which implies the surface charging-induced etching stops. The etching, however, slightly increased with the increasing ion flux from the reference condition, revealing the surface charge removal accompanied with conducting fluorocarbon film formation by heavy radicals. In addition, the entrance width of an amorphous carbon layer (ACL) mask enlarges with increasing ion energy, whereas it relatively remains constant with that of ion energy. These findings can be utilized to optimize the SiO${}_{2}$ etching process in high-aspect ratio etching applications.

## 1. Introduction

Plasma, which is composed of charged and neutral particles, has been widely used in material processing due to its chemically reactive species and energetic ions, which can activate and modify the surface of materials [1]. These characteristics have made plasma processing a significant tool in semiconductor fabrication, particularly in high-aspect ratio (HAR) etching for creating three-dimensional transistor structures and improving transistor stacking [2,3,4].

As the feature size shrinks to nano and atomic scales, and the layer stacking increases, severe challenges emerge in HAR etching, including notching, bowing, twisting, and tapering, and aspect ratio-dependent etching [4,5]. To overcome these issues, multi-frequency modulation, voltage wave tailoring, and pulse-modulated methods have been developed for precise control of ion energy and ion-to-neutral flux [6,7,8,9]. Furthermore, processing has become more complicated; the number of processing steps increases and complex gas mixtures are employed. Due to its complexity, the exact etching mechanism has yet to be clearly understood; etching depends on various internal parameters of plasma and chemical species, which are difficult to measure and control due to the complex nature of plasma. As a result, most experimental studies have analyzed the effects of external parameters such as gas composition, bias voltage, and power on HAR etching characteristics [2,3,10,11,12].

Alternatively, several computational studies have investigated the etching mechanism in HAR features, considering internal parameters. For instance, Huang et al. [13] investigated HAR etching with a C${}_{4}$F${}_{8}$/Ar/O${}_{2}$ mixture based on a hybrid plasma equipment model (HPEM) and Monte Carlo feature profile model (MCFPM). They found that hot neutrals formed by the charge neutralization process of energetic ions with the side wall inside the HAR trench can reach the deep etch front through the Knudsen transport and become the main etchant. On the other hand, radical species entering the trench entrance cannot reach the deep etch front due to its isotropic nature and their high reactivity with the side wall. Wang et al. [14] found an etching mechanism transition from fluorocarbon (FC) film deposition to chemical sputtering in a C${}_{4}$F${}_{6}$/Ar/O${}_{2}$ gas mixture environment through HPEM and MCFPM simulation. Kwon et al. [15] investigated the role of heavy ion impact on the feature-to-feature profile of SiO${}_{2}$ by using a multi-scale computer simulation. They found that heavy ion sputtering reduces the feature-to-feature distortion. Recently, Jeong et al. [16] experimentally established well-controlled regimes of ion and radicals fluxes in C${}_{4}$F${}_{8}$/Ar plasma by varying the C${}_{4}$F${}_{8}$ fraction and using pulse-modulated radio-frequency power, and they investigated etching characteristics at each regime. They found that the neutral-to-ion flux ratio is the key factor for etching dynamics. At a low neutral-to-ion flux ratio, the etching rate increases with the increasing of the flux ratio due to enhanced chemical reactions on the etch front, whereas at a high neutral-to-ion flux ratio, the etching rate decreases with the increasing of the flux ratio due to the thick FC film formation. Furthermore, their results exhibited that ion transport toward the etch front inside the HAR feature is a significant factor for understanding the etching mechanism.

Because experimental and computational studies have revealed that ions inside the HAR feature play a significant role in HAR SiO${}_{2}$ etching [13,14,15,16], most simulation studies have focused on the validation of technologies for individual control of ion flux and energy. For instance, Kim et al. [17] proved the capability of the individual control of ion flux and energy distribution by varying dual-frequency voltages through a two-dimensional particle-in-cell Monte Carlo collisions (PIC-MCC) simulation. Schulze et al. [18] proved the voltage pulse wave tailoring technology for individual control of the ion flux and energy through a two-dimensional PIC-MCC simulation in dual-frequency voltage sources. In addition, most experimental studies analyzed the coupled influence of ion flux and energy for etching based on the ion parameter measurement [3,10,19]. However, the detailed analysis on the separate influence of ion flux and energy on SiO${}_{2}$ etching in the HAR feature based on the individual control and measurement of ion parameters has yet to be investigated. In this study, we established control regimes for ion parameters, the ion energy and flux, at dual-frequency capacitively coupled C${}_{4}$F${}_{8}$/Ar plasma and elucidated their contribution on HAR SiO${}_{2}$ etching characteristics.

This paper is organized as follows: In Section 2, we describe the experimental setup and the individual control regime of ion energy and flux. Section 3 provides the experimental results and detailed analysis. In Section 4, we summarize the findings of this study.

## 2. Experiment Setup, Measurement Methods, and Volume-Ag Plasma Model

### 2.1. Dual-Frequency Capacitively Coupled Plasma Source

**Figure 1:**
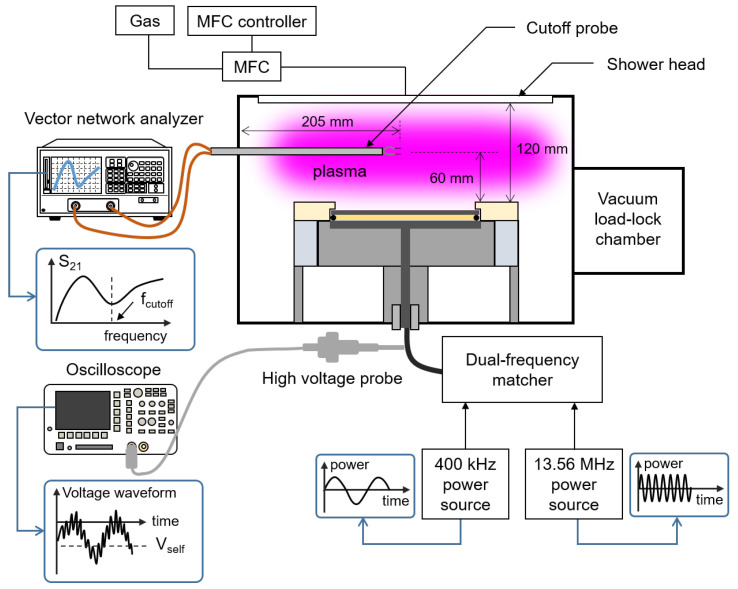
Schematic diagram of a dual-frequency capacitively coupled Ar/C${}_{4}$F${}_{8}$ plasma source.

We employed a dual-frequency capacitively coupled Ar/C${}_{4}$F${}_{8}$ plasma source as shown in Figure 1. The experimental setup is the same as that in [16] except the additional power source. Discharge power from 13.56 MHz (AE paramount, Advanced energy Inc., Denver, CO, USA) and 400 kHz power sources (AE paramount, Advanced energy Inc., Denver, CO, USA) is applied to the powered electrode with a diameter of 200 mm through a dual-frequency matcher (Pathfinder Dual, Plasmart, Daejeon, Republic of Korea). The discharge gap is 120 mm. Processing gases, C${}_{4}$F${}_{8}$ and Ar, are injected into a vacuum chamber with radius of 400 mm through a shower head. The flow rate of C${}_{4}$F${}_{8}$ and Argon is 80 and 20 standard cubic centimeter per minute (sccm), respectively, controlled by a mass flow controller (MFC) (Linetech, Daejeon, Republic of Korea). A turbomolecular pump (TG1003, Osaka vacuum, Ltd., Osaka, Japan) accompanied with a dry pump (ADP122, Alcatel, Aβlar, Germany) draw the gases. The chamber pressure is maintained as 20 mTorr, which is regulated by adjusting a throttle valve (Atovac, Gyeonggi-do, Republic of Korea).

The powered electrode consists of top and bottom parts. As the high power is applied in the whole powered electrode, coolant flows through the bottom of the powered electrode. Furthermore, high-pressure helium gas is injected to the electrode for improving thermal contact between the top and bottom of the powered electrode. Here, a wafer sample is located on the top of the powered electrode.

### 2.2. Wafer Sample

**Figure 2:**
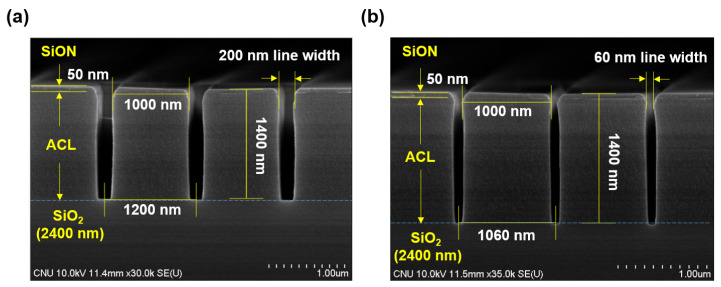
Scanning electron microscope (SEM) images of a cross-sectional view of a patterned wafer sample for (**a**) 200 nm and (**b**) 60 nm line widths.

A coupon wafer with 12 mm × 7 mm is located at the center of the electrode through a vacuum load-lock chamber. Figure 2 shows scanning electron microscope (SEM) images of a cross section of the wafer before etching. There is a patterned amorphous carbon layer (ACL) mask with thickness of 1400 nm, which is widely used in HAR etching as a mask [16,20]. It has a trench pattern with various line widths ranging from 60 nm to 200 nm. At the top of the ACL mask, there is a thin SiON layer with thickness of 50 nm. At the bottom of the ACL mask, there is a SiO${}_{2}$ layer with thickness of 2400 nm deposited through plasma-enhanced tetra ethylene ortho silicate (PE-TEOS) processing. At the bottom of the SiO${}_{2}$ layer, there is a Si substrate.

After etching, the sample is extracted from the chamber and then moved to SEM instrument.

### 2.3. Ion Flux and Energy Measurement Method

In plasma diagnostics, ion parameters such as ion flux ($\Gamma_{\mathrm{ion}}$) and energy ($E_{\mathrm{ion}}$) can be determined with a retarded-field energy analyzer (RFEA) by measuring ion conduction current and deriving ion energy distribution function. However, in processing plasma, the RFEA is not applicable due to polymeric film deposition, leading to block the conduction current. Furthermore, measuring ion parameters incident on the powered electrode (PE) with the RFEA suffers from huge noises induced by the PE. Alternatively, we estimate the ion flux incident on the PE ($\Gamma_{\mathrm{ion}}^{\mathrm{PE}}$), where a wafer sample locates, by measuring bulk electron density ($n_{e}$) [2,3].

Following is an explanation of this method to estimate the $\Gamma_{\mathrm{ion}}^{\mathrm{PE}}$ with the $n_{e}$. In electronegative plasma as with C${}_{4}$F${}_{8}$ plasma, the $n_{e}$ is the same with the sum of positive ion densities at the plasma–sheath boundary (PSB) due to quasi-neutral character of plasma as

(1) $$n_{e}=\sum_{\mathrm{species}}n_{\mathrm{ion}}^{\mathrm{species}}.$$

Here, the sheath is the space-charge region covering plasma where ions are accelerated to the material surface. Negative ions are not taken into account in Equation (1) because the electronegative core where negative ions are confined is restricted inside the plasma bulk [21]. Specifically, the negative ions are under Maxwellian distribution with an ion temperature of T${}_{i}$, which can be assumed as room temperature (T${}_{i}$ = 0.026 eV) in low-temperature plasma [22], and thus, most negative ions cannot escape the plasma bulk because the ambipolar potential in the bulk is in the order of a few eV. Hence, only electrons and positive ions exist at the PSB, and Equation (1) is valid. The $\Gamma_{\mathrm{ion}}^{\mathrm{PSB}}$ is defined as

(2) $$\Gamma_{\mathrm{ion}}^{\mathrm{PSB}}=\sum_{\mathrm{species}}n_{\mathrm{ion}}^{\mathrm{species}}u_{B}^{\mathrm{species}}$$

where $u_{B}^{\mathrm{species}}\left(=\sqrt{eT_{e}/M_{\mathrm{ion}}^{\mathrm{species}}}\right)$ is the Bohm velocity of ion, *e* is the elementary charge, T${}_{e}$ is the electron temperature, and $M_{\mathrm{ion}}^{\mathrm{species}}$ is the mass of the ion [21]. As the Bohm velocity has a root power for the $M_{\mathrm{ion}}^{\mathrm{species}}$ and the mass of dominant ion species varies from 40 amu (Ar) to 131 amu (C${}_{3}$F${}_{5}$) [13], where amu is atomic mass unit (1 amu = 1.66 $\times \;10^{-27}$ kg), it can be assumed as a constant and we can take the Bohm velocity out of the summation in Equation (2). Then, the $\Gamma_{\mathrm{ion}}^{\mathrm{PSB}}$ is proportional to the sum of the positive ion densities ($\sum n_{\mathrm{ion}}^{\mathrm{species}}$), and therefore to the $n_{e}$ as

(3) $$\Gamma_{\mathrm{ion}}^{\mathrm{PSB}}\propto n_{e}$$

Furthermore, the sheath in 20 mTorr can be assumed as a collisionless environment, and the ion flux is conserved, ensuring that the ion flux at the PSB is the same as that on the PE as

(4) $$\Gamma_{\mathrm{ion}}^{\mathrm{PSB}}=\Gamma_{\mathrm{ion}}^{\mathrm{PE}},$$

and thus,

(5) $$\Gamma_{\mathrm{ion}}^{\mathrm{PE}}\propto n_{e}.$$

We measured the $n_{e}$ with the cutoff probe at the chamber center, which is a precise tool for electron density measurement applicable to plasma processing [23,24]. The detailed principle is well-described elsewhere [23]. The cutoff probe utilizes small-power microwaves ranging from a few MHz to GHz and measures the cutoff frequency ($f_{\mathrm{cutoff}}$) in a transmission microwave frequency spectrum. In a low-pressure condition, the $f_{\mathrm{cutoff}}$ has a relationship with the $n_{e}$ as [23]

(6) $$f_{\mathrm{cutoff}}=\sqrt{\frac{e^{2}n_{e}}{\epsilon_{0}m_{e}}}$$

where $\epsilon_{0}$ is the permittivity in vacuum and $m_{e}$ is the electron mass. Here, we measure the transmission microwave frequency spectrum with a vector network analyzer (E5071B, Agilent technologies Inc., Santa Clara, CA, USA).

In addition to the $n_{e}$, we can estimate the $E_{\mathrm{ion}}$ by measuring the self-bias voltage ($V_{\mathrm{self}}$). Following is the explanation of this method. The ions are accelerated by the electric field in the sheath and strike the wafer sample with kinetic energy, $E_{\mathrm{ion}}$. As the sheath oscillates, the $E_{\mathrm{ion}}$ is proportional to the time-averaged value of the voltage difference between the plasma potential ($V_{p}\left(t\right)$) and the powered electrode voltage ($V_{\mathrm{PE}}\left(t\right)$) [21], that is,

(7) $$E_{\mathrm{ion}}\propto \frac{1}{T}\int_{0}^{T}e(V_{p}\left(t\right)-V_{\mathrm{PE}}\left(t\right))dt,$$

where *T* is the period of the low-deriving frequency. In this system, the peak to peak of the $V_{\mathrm{PE}}\left(t\right)$ is a few kV and that of the $V_{p}\left(t\right)$ is to the order of ten volts in a capacitively coupled plasma [21], and thus, we can neglect the $V_{p}\left(t\right)$ in Equation (7). The $V_{\mathrm{self}}$ is defined as the time-averaged value of the powered electrode voltage, that is,

(8) $$V_{\mathrm{self}}=\frac{1}{T}\int_{0}^{T}V_{\mathrm{PE}}\left(t\right)dt.$$

Thus, the $E_{\mathrm{ion}}$ is proportional to the $V_{\mathrm{self}}$ [25,26], that is,

(9) $$E_{\mathrm{ion}}\propto V_{\mathrm{self}}.$$

To derive the $V_{\mathrm{self}}$, we measured a voltage waveform with a high-voltage probe (P5100A, Tektronix Inc., Beaverton, OR, USA) and an oscilloscope (TDS3052B, Tektronix Inc., Beaverton, OR, USA) as shown in Figure 1.

### 2.4. Ion Energy and Flux-Controlled Regimes

To control the ion energy and flux, we adjusted the dual-frequency powers. For the ion flux-controlled condition, we adjust 13.56 MHz powers for controlling the $n_{e}$ and 400 kHz powers for fixing the $V_{\mathrm{self}}$. Similarly, for the ion energy-controlled condition, we adjust 400 kHz powers for controlling the $V_{\mathrm{self}}$ and 13.56 MHz power for fixing the $n_{e}$.

**Figure 3:**
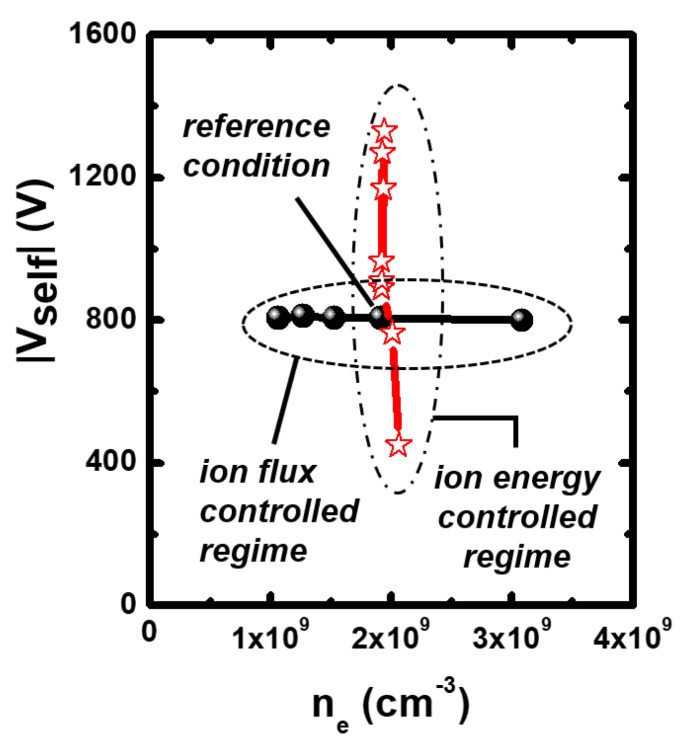
Measured self-bias voltages (V${}_{\mathrm{self}}$s) and electron densities ($n_{e}$) for ion flux- and ion energy-controlled regimes.

Figure 3 shows the variation in the $n_{e}$ at fixed $V_{\mathrm{self}}$, indicating the ion flux-controlled regime, and the variation in $V_{\mathrm{self}}$ at the fixed $n_{e}$, indicating the ion energy-controlled regime. Hence, we established the ion flux- and the ion energy-controlled regimes.

### 2.5. Volume-Averaged Plasma Model

As the electron density increases, the radicals density inevitably changes because electrons dominate the radicals’ production reactions [2,3,16,21]. The precise measurement of the radicals density is a great challenge in plasma processing. Several studies used optical emission spectroscopy [27,28] and a quadrupole mass spectrometer [2,29] for estimating radicals densities, but its accuracy remains questionable. Alternatively, a volume-averaged model was used for analyzing plasma chemistry in a complex gas mixture [30,31,32]. We developed an in-house volume-averaged model code for C${}_{4}$F${}_{8}$/Ar plasma.

The model solves particle and power balance equations. The particle balance equation for each particle besides electrons, shown in Table 1, is as [33]

(10) $$\frac{dn_{j}}{dt}=\frac{Q}{V_{\mathrm{vol}}}-\frac{1}{\tau }n_{j}+\sum_{i,k}K_{i,k}\left(T_{e}\right)n_{i}n_{k}-\frac{\Gamma_{j}A_{\mathrm{eff}}}{V_{\mathrm{vol}}}$$

where $n_{j}$ is the particle density for *j* species, $V_{\mathrm{vol}}$ is the plasma volume, and *Q* is the input flow rate of feedstock gas, for instance, as for CF${}_{x}$ and C${}_{x}$F${}_{y}$ species, *Q* is the flow rate of C${}_{4}$F${}_{8}$, 80 sccm, and as for Ar species, *Q* is that of Ar, 20 sccm, τ is the residence time, $K_{i,k}\left(T_{e}\right)$ is the reaction rate constant, $\Gamma_{j}$ is the particle flux to the wall, and $A_{\mathrm{eff}}$ is the effective area of plasma. The τ is defined as [21]

(11) $$\tau =pV_{\mathrm{vol}}/Q_{\mathrm{tot}}$$

where $T_{g}$ is the gas temperature, *p* is the chamber pressure, $Q_{\mathrm{tot}}$ is the total flow rate, and the $A_{\mathrm{eff}}$ is defined as

(12) $$A_{\mathrm{eff}}=2\pi R^{2}h_{L}+2\pi RLh_{R}$$

where *R* is the plasma radius, and *L* is the discharge gap. Here, $h_{L}$ and $h_{R}$ are the edge-to-center ratio defined as [21]

(13) $$h_{L}=\frac{0.86\left(1+\frac{3\alpha }{\gamma }\right)}{\left(1+\alpha \right)\sqrt{3+\frac{L}{2\lambda_{i}}}},$$

and

(14) $$h_{R}=\frac{0.80\left(1+\frac{3\alpha }{\gamma }\right)}{\left(1+\alpha \right)\sqrt{4+\frac{R}{\lambda_{i}}}},$$

where α(=$\sum n_{-}/n_{e})$ is the electronegativity, $\sum n_{-}$ is the total negative ion density, γ(=$T_{e}/T_{i})$ is the temperature ratio, T${}_{i}$ is the ion temperature, and $\lambda_{i}$ is the mean-free path. It is noted that electron density is governed not by the particle balance equations but by the quasi-neutrality condition of plasma as

(15) $$n_{e}=\sum_{l}n_{l}-\sum_{m}n_{m}$$

where *l* and *m* mean the positive and negative ion species, respectively.

To calculate the reaction rate, we referred to various reactions for C${}_{4}$F${}_{8}$ reactions [32,33,34], Argon [35], and C${}_{4}$F${}_{8}$-Ar reactions [36] combined into this model. Detailed reaction tables are represented in Table A1,Table A2,Table A3,Table A4,Table A5,Table A6,Table A7 and Table A8.

The model solves the power balance equation for electrons as

(16) $$\frac{d}{dt}\left(\frac{3}{2}en_{e}T_{e}\right)=\frac{1}{V_{\mathrm{vol}}}\left(P_{\mathrm{abs}}-P_{\mathrm{loss}}\right)$$

where $P_{\mathrm{abs}}$ is the input power and $P_{\mathrm{loss}}$ is the loss power of electrons, defined as

(17) $$P_{\mathrm{loss}}=P_{c}+P_{\mathrm{ei}}$$

where $P_{c}$ is the electron–ion pair creation loss, defined as

(18) $$P_{c}=en_{e}V_{\mathrm{vol}}\sum_{j}n_{j}\left(\sum_{i}K_{\mathrm{dis}}^{j,i}E_{\mathrm{dis}}^{j,i}+\sum_{k}K_{\mathrm{iz}}^{j,k}E_{\mathrm{iz}}^{j,k}+K_{\mathrm{el}}\frac{3m_{e}}{M_{j}}\left(T_{e}-\frac{k_{B}T_{g}}{e}\right)\right),$$

and $P_{\mathrm{ei}}$ is the ion and electron kinetic power losses, defined as

(19) $$P_{\mathrm{ei}}=e\left(E_{\mathrm{ion}}+E_{e}\right)A_{\mathrm{eff}}\sum_{j=\mathrm{ion}}n_{j}u_{j}$$

where $E_{\mathrm{ion}}($=$V_{f}+T_{e}/\left(2\left(1+\alpha \right)\left(1+\alpha \gamma \right)\right)$ is the ion kinetic energy and $E_{e}($=$2T_{e})$ is the average kinetic energy of electrons coming out of the plasma. The $V_{f}$ is the floating potential derived as the floating condition as

(20) $$\sum_{l}n_{l}u_{l}=\sum_{m}n_{m}v_{m}\exp\left(-\frac{V_{f}}{T_{m}}\right)$$

where *l* and *m* mean the positive and negative ion species, respectively, $v_{m}($=$\sqrt{8eT_{m}/\left(\pi M_{m}\right)})$ is the thermal velocity, $T_{m}$ is the negative ion temperature, and $M_{m}$ is the negative ion mass.

## 3. Results and Discussion

### 3.1. Ion Energy Contribution on Etching Characteristics

**Figure 4:**
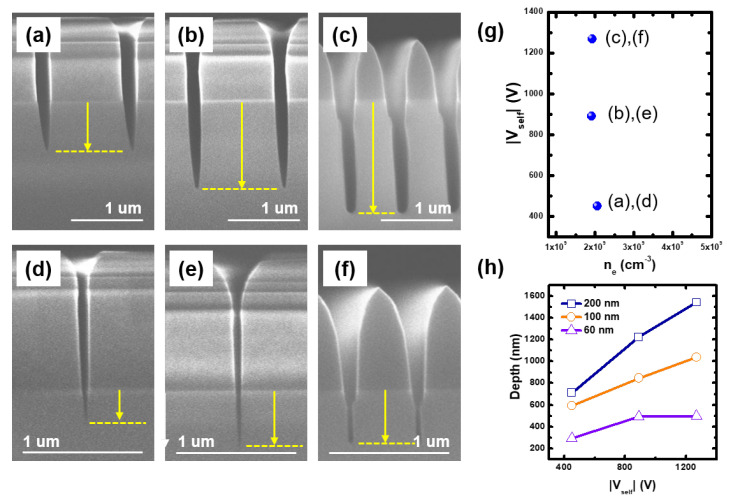
Scanning electron microscope (SEM) images for (**a**–**c**) 200 nm and (**d**–**f**) 60 nm line widths at self-bias voltages ($V_{\mathrm{self}}$) of (**a**,**d**) 450 V, (**b**,**e**) 890 V, and (**c**,**f**) 1270 V with fixed electron density ($n_{e}$) about 2.0 × 10${}^{9}$ cm${}^{-3}$. (**g**) Measured V${}_{\mathrm{self}}$ and electron densities at each condition ((**a**,**d**), (**b**,**e**), and (**c**,**f**)), and (**h**) measured etch depth over V${}_{\mathrm{self}}$. Here, the tilted profiles and stripe patterns are caused by the set-up and charge-up during SEM image capture, respectively.

At first, we investigated the contribution of ion energy on etching characteristics. Figure 4a–c and Figure 4d–f represent SEM images at the 200 nm and 60 nm line widths, respectively, with various $V_{\mathrm{self}}$s. Here, the etching time was fixed as 20 min for all cases. In these conditions, the electron density was fixed as shown in Figure 4g. At the 200 nm line width, the etch depth, marked as the arrow and dashed line in the SEM images, deepens with the $V_{\mathrm{self}}$. Because the ion flux is fixed, the result ensures that the ion energy can enhance the etching process.

However, it is noted that this enhancement deteriorates with the narrowing of the line width as shown in Figure 4h; the slope decreases as the line width narrows. There are two possible causes of the decrease in the ion bombardment effect: thick FC film formation by C${}_{4}$F${}_{8}$ fragments, such as C${}_{x}$F${}_{y}$, and ion angle distribution. The FC film plays a role in protecting the SiO${}_{2}$ from energetic ions [37,38]. However, a thin FC film (a few nano-meters) would be maintained during the etching process in this $V_{\mathrm{self}}$ range; as the ion energy is high enough to make a high sputtering yield, this compensates the FC film deposition rate and a thin FC film forms [38,39]. In addition, the amount of C${}_{x}$F${}_{y}$ species entering the trench decreases with the narrowing of the line width due to the shrinking of the trench entrance. It leads to the drop in the etch depth at the same ion energy shown in Figure 4h. Hence, the thick FC film formation is not realistic in this condition. Then, the ion angle distribution, the second reason, dominates the decrease in the enhancement. Ions accelerated in the sheath are able to collide with background gases, and their incident angle to the trench has an angle distribution depending on the pressure and sheath width [21]. Ions with a large incident angle are transported to the etch front inside the trench through multiple reflections on the side wall and then strike the etch front with lower kinetic energy, causing the decrease in the ion bombardment enhancement [40].

In addition, the etch stop occurs at the 60 nm line width despite the ion energy elevation as shown in Figure 4h. It implies that the increase in ion energy is not a perfect solution for the optimization of the HAR SiO${}_{2}$ etching process. The etch stop in the HAR structure results from the charge accumulation on the narrow SiO${}_{2}$ structure, leading to ion repelling and insufficient radicals provision. As the trench depth deepens, the charge accumulation on the SiO${}_{2}$ surface increases. Moreover, the electric conductivity of the FC film decreases, which enhances the charge accumulation on the trench surface [41]. As for radicals provision, in this experiment, sufficient radicals species enter the trench because the trench width widens with increasing ion energy, as shown in Figure 4d–f. Furthermore, the radicals compositions remain unchanged due to constant $n_{e}$ with increasing ion energy. Hence, it is noted that ion energy enhances the etching process with C${}_{4}$F${}_{8}$/Ar, but in the HAR structure, the charge accumulation restricts its effect even in a sufficient radicals environment.

The trench entrance width enlarges with increasing ion energy regardless of the trench width. This entrance opening results from the angular dependence of the sputtering yield of the ACL. The sputtering yield has a peak near 70${}^{\circ }$ of the incidence angle; it is formed by the balance between the increase in energy deposited on the surface by the incident ion and the decrease in the depth traveled by the sputtered atom [42]. As the edge of the trench entrance is rounded as shown in Figure 2, the etching rate of the entrance edge is larger than that of the side wall, leading to the entrance opening. Furthermore, the etching rate of the ACL mask abruptly elevates at 1270 V, whereas that of SiO${}_{2}$ monotonically increases. The elevation differs with the general trend of etching yield dependence on incident ion energy; the etching yield is proportional to a square root of the ion energy [43]. As it is beyond the scope of this study, we retain it as future work. In summary, increasing ion energy in high V${}_{\mathrm{self}}$ allows to enlarge the feature size through the ACL mask opening and to lower the etching selectivity.

### 3.2. Ion Flux Contribution on Etching Characteristics

**Figure 5:**
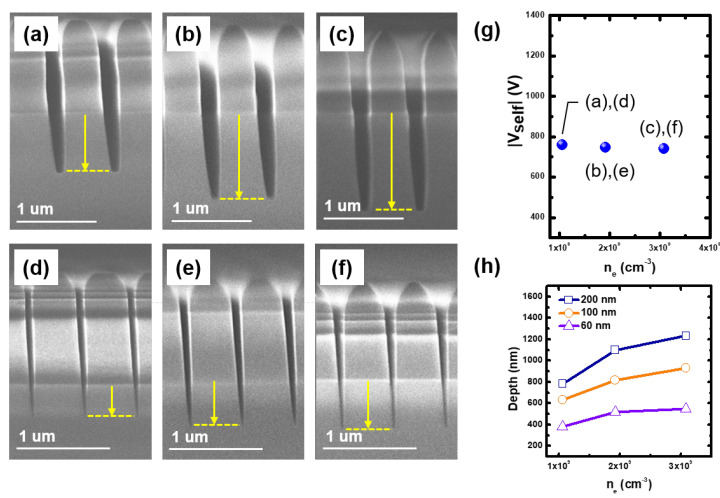
Scanning electron microscope (SEM) images for (**a**–**c**) 200 nm and (**d**–**f**) 60 nm line widths at (**a**,**d**) 1.1 × 10${}^{09}$ cm${}^{-3}$, (**b**,**e**) 1.9 × 10${}^{09}$ cm${}^{-3}$, and (**c**,**f**) 3.1 × 10${}^{09}$ cm${}^{-3}$, with the fixed self-bias voltage about 740 V. (**g**) Measured V${}_{\mathrm{self}}$ and electron densities at each condition ((**a**,**d**), (**b**,**e**), and (**c**,**f**)), and (**h**) measured etch depth over n${}_{e}$. Here, the tilted profiles and stripe patterns are caused by the set-up and charge-up during SEM image capture, respectively.

**Figure 6:**
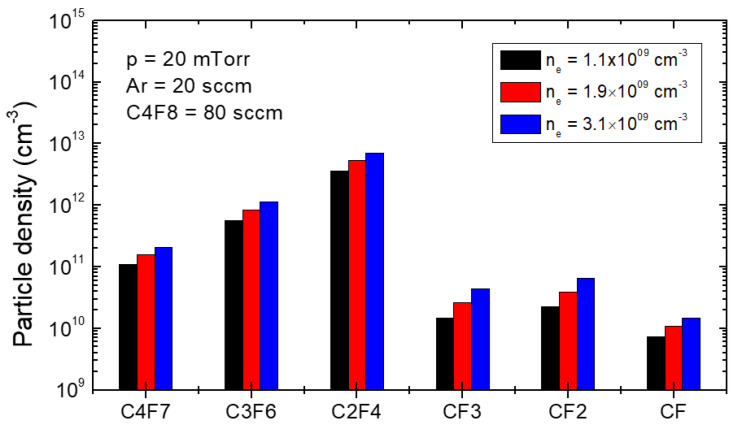
Radicals densities (C${}_{4}$F${}_{7}$, C${}_{3}$F${}_{6}$, C${}_{2}$F${}_{4}$, CF${}_{3}$, CF${}_{2}$, CF) at different electron densities ($n_{e}$s) calculated by an in-house volume-averaged plasma model.

As for the ion flux contribution on the etching characteristics, Figure 5a–c and Figure 5d–f represent SEM images at 200 nm and 60 nm line widths, respectively, with various $n_{e}$s. Here, the etching time was fixed as 20 min for all cases. In this condition, the $V_{\mathrm{self}}$ was fixed as shown in Figure 5g. As shown in Figure 5h, the etch depth increases with increasing ion flux. Because the ion energy is fixed, the result ensures that the increase in the ion flux enhances the etching process. However, the slope drops over the ion flux. It results from the FC film formation due to the increase in the radicals density. Figure 6 shows the calculated radicals densities at various electron densities in the same pressure and gas mixture condition. Here, the electron density was adjusted with increasing discharge power. In SiO${}_{2}$ etching with FC plasma, heavy radicals (C${}_{x}$F${}_{y}$) play a role in the formation of a thick FC film [38,39]. As shown in Figure 6, heavy radicals are larger than light radicals (CF${}_{x}$), and thus, a thick FC film deposition would be formed with increasing electron density.

Moreover, it is noted that the etch stop deteriorates; the etch stop occurs at a 500 nm etch depth in the ion energy-controlled regime, but the etch depth slightly increases with the increasing of the ion flux as shown in Figure 5h. The etch stop relaxation could correspond to the reduction in charge accumulation. The thicker FC film would prohibit charge accumulation inside the trench because it has conductivity, and thus, the etch stop can be moderated.

As for the trench width, it remains preserved with the increasing of the ion flux compared to the case of the ion energy increase. It results from the increase in the FC film, which plays a role in a passivation layer for the ACL. Furthermore, because the increase in the etching rate is monotonic, the etching selectivity remains constant. Hence, we can conclude that the increase in the ion flux allows to elevate the etching rate with preserving both the feature size and etching selectivity by providing a passivation layer on the ACL mask.

### 3.3. Comparison of Individual Contributions of Ion Flux and Energy on SiO${}_{2}$ Etching Rate

**Figure 7:**
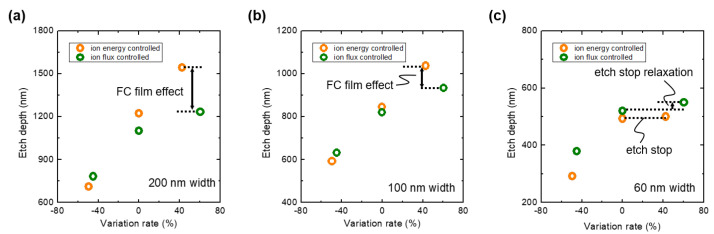
Etch depth over variation rate of ion flux and energy in ion flux- and energy-controlled regimes at (**a**) 200 nm, (**b**) 100 nm, and (**c**) 60 nm line widths.

To compare the contributions of the ion energy and flux, the etch depth over the parameter variation rate based on Figure 4 and Figure 5 is represented in Figure 7. Here, the zero in each condition is the reference condition shown in Figure 3. At 200 nm and 100 nm trench widths, one can find that the increase in the ion energy enhances the etching rate more than that in the ion flux with the same variation rates; the slope of the ion energy-controlled regimes is larger than that of the ion flux-controlled regime.

In addition, based on Figure 7, one can figure out the FC film effect. There is a clear gap between the etch depth of the ion energy and ion flux controlled at about a 40% variation rate for the 200 nm and 100 nm trench widths, as shown in Figure 7a,b. It implies the FC film effect is formed by heavy radicals. As the FC film plays a role in a passivation layer, it allows the decrease in the etch depth. Specifically, because the line width is proportional to the entrance area of the trench, the amount of radicals entering the trench is larger at the 200 nm width than the 100 nm width. As a result, it leads to the increase in the difference in the etch depth at the 200 nm width.

Moreover, the etch stop at the reference condition at the 60 nm trench width and its relaxation by increasing the ion flux can be clearly observed in Figure 7c. It implies that the increase in heavy radicals production is more effective to moderate the etch stop phenomenon than the increase in the ion energy in FC plasma.

## 4. Conclusions

This study investigated the contribution of ion energy and flux on HAR SiO${}_{2}$ etching characteristics. We established the process window where the ion flux and energy are individually controlled. As a result, ion energy enhances the etching process, but in a high-aspect ratio structure, charge accumulation restricts its effect even in a sufficient radicals environment. We found that this charge accumulation can be released with increasing radicals species, leading to the slight increase in FC film formation having conductivity. Here, we estimated the density of radicals species such as C${}_{x}$F${}_{y}$ and CF${}_{x}$ with an in-house volume-averaged plasma model. The model exhibits the increase in radicals density, implying the increase in the FC film thickness. Furthermore, we found that the increase in the ion energy enhances the etching more than that in the ion flux with the same variation rate.

## Figures and Tables

**Table 1:** Species included in the volume-averaged plasma model, besides the electron. Here, CF* and CF${}_{2}$* mean the excited species of CF and CF${}_{2}$, respectively.

Type	Species
C species	C, C${}^{+}$
F species	F, F${}^{+}$, F${}^{-}$, F${}_{2}$, F${}_{2}^{+}$, F${}_{2}^{-}$
CF${}_{a}$ species	CF, CF*, CF${}^{+}$, CF${}_{2}$, CF${}_{2}$*, CF${}_{2}^{+}$, CF${}_{3}$, CF${}_{3}^{+}$, CF${}_{3}^{-}$, CF${}_{4}$
C${}_{2}$F${}_{b}$ species	C${}_{2}$F${}_{3}$, C${}_{2}$F${}_{3}^{+}$, C${}_{2}$F${}_{4}$, C${}_{2}$F${}_{4}^{+}$, C${}_{2}$F${}_{5}$, C${}_{2}$F${}_{5}^{+}$, C${}_{2}$F${}_{6}$
C${}_{3}$F${}_{c}$ species	C${}_{3}$F${}_{5}$, C${}_{3}$F${}_{5}^{+}$, C${}_{3}$F${}_{6}$, C${}_{3}$F${}_{6}^{+}$, C${}_{3}$F${}_{7}$, C${}_{3}$F${}_{7}^{+}$
C${}_{4}$F${}_{d}$ species	C${}_{4}$F${}_{7}$, C${}_{4}$F${}_{7}^{+}$, C${}_{4}$F${}_{8}$, C${}_{4}$F${}_{8}^{-}$
Ar species	Ar, Ar${}^{m}$, Ar${}^{r}$, Ar(4p), Ar${}^{+}$

## Data Availability

The data presented in this study are available on request from the corresponding author.

## Appendix A

Reactions used in a volume-averaged plasma model are in Table A1,Table A2,Table A3,Table A4,Table A5,Table A6,Table A7 and Table A8.

**Table A1:** Argon-electron and Argon-Argon reaction set included in a volume-averaged plasma model.

	Reaction	Rate Constant	Unit	Reference
(R1)	e+Ar→e+Ar${}^{m}$	9.90 $\times \;10^{-16}T_{e}^{-0.08}\exp\left(-11.72/T_{e}\right)$	m${}^{3}$s${}^{-1}$	[35]
(R2)	e+Ar→e+Ar${}^{r}$	4.03 $\times \;10^{-15}T_{e}^{0.45}\exp\left(-12.12/T_{e}\right)$	m${}^{3}$s${}^{-1}$	[35]
(R3)	e+Ar→e+Ar(4p)	9.26 $\times \;10^{-15}T_{e}^{-0.06}\exp\left(-14.24/T_{e}\right)$	m${}^{3}$s${}^{-1}$	[35]
(R4)	e+Ar→2e+Ar${}^{+}$	2.39 $\times \;10^{-14}T_{e}^{0.57}\exp\left(-17.43/T_{e}\right)$	m${}^{3}$s${}^{-1}$	[35]
(R5)	e+Ar${}^{m}\to$e+Ar	2.25 $\times \;10^{-16}T_{e}^{-0.17}\exp\left(-1.65/T_{e}\right)$	m${}^{3}$s${}^{-1}$	[35]
(R6)	e+Ar${}^{m}\to$e+Ar${}^{s}$	3.70 $\times \;10^{-13}$	m${}^{3}$s${}^{-1}$	[35]
(R7)	e+Ar${}^{m}\to$e+Ar(4p)	2.48 $\times \;10^{-12}T_{e}^{-0.16}\exp\left(-1.88/T_{e}\right)$	m${}^{3}$s${}^{-1}$	[35]
(R8)	e+Ar${}^{m}\to$2e+Ar${}^{+}$	2.99 $\times \;10^{-13}T_{e}^{0.22}\exp\left(-4.73/T_{e}\right)$	m${}^{3}$s${}^{-1}$	[35]
(R9)	e+Ar${}^{r}\to$e+Ar	6.82 $\times \;10^{-16}T_{e}^{0.44}\exp\left(-0.43/T_{e}\right)$	m${}^{3}$s${}^{-1}$	[35]
(R10)	e+Ar${}^{r}\to$e+Ar${}^{m}$	9.10 $\times \;10^{-13}$	m${}^{3}$s${}^{-1}$	[35]
(R11)	e+Ar${}^{r}\to$e+Ar(4p)	2.48 $\times \;10^{-12}T_{e}^{-0.16}\exp\left(-1.79/T_{e}\right)$	m${}^{3}$s${}^{-1}$	[35]
(R12)	e+Ar${}^{r}\to$2e+Ar${}^{+}$	3.28 $\times \;10^{-13}T_{e}^{0.21}\exp\left(-4.51/T_{e}\right)$	m${}^{3}$s${}^{-1}$	[35]
(R13)	e+Ar(4p)→e+Ar	2.97 $\times \;10^{-16}T_{e}^{-0.11}\exp\left(-1.38/T_{e}\right)$	m${}^{3}$s${}^{-1}$	[35]
(R14)	e+Ar(4p)→e+Ar${}^{m}$	4.16 $\times \;10^{-13}T_{e}^{-0.17}\exp\left(-0.32/T_{e}\right)$	m${}^{3}$s${}^{-1}$	[35]
(R15)	e+Ar(4p)→e+Ar${}^{r}$	4.16 $\times \;10^{-13}T_{e}^{-0.17}\exp\left(-0.32/T_{e}\right)$	m${}^{3}$s${}^{-1}$	[35]
(R16)	e+Ar(4p)→2e+Ar${}^{+}$	1.23 $\times \;10^{-12}T_{e}^{0.25}\exp\left(-3.71/T_{e}\right)$	m${}^{3}$s${}^{-1}$	[35]
(R17)	Ar${}^{m}$+Ar${}^{m}\to$e+Ar${}^{+}$+Ar	6.40 $\times \;10^{-16}$	m${}^{3}$s${}^{-1}$	[35]
(R18)	Ar${}^{m}$+Ar${}^{r}\to$e+Ar${}^{+}$+Ar	2.10 $\times \;10^{-15}$	m${}^{3}$s${}^{-1}$	[35]
(R19)	Ar${}^{r}$+Ar${}^{r}\to$e+Ar${}^{+}$+Ar	5.00 $\times \;10^{-16}$	m${}^{3}$s${}^{-1}$	[35]
(R20)	Ar(4p)+Ar(4p)→e+Ar${}^{+}$+Ar	5.00 $\times \;10^{-16}$	m${}^{3}$s${}^{-1}$	[35]
(R21)	Ar(4p)+Ar${}^{m}\to$e+Ar${}^{+}$+Ar	5.00 $\times \;10^{-16}$	m${}^{3}$s${}^{-1}$	[35]
(R22)	Ar(4p)+Ar${}^{r}\to$e+Ar${}^{+}$+Ar	5.00 $\times \;10^{-16}$	m${}^{3}$s${}^{-1}$	[35]
(R22)	Ar${}^{r}\to$ Ar+hν	1.00 $\times \;10^{5}$	s${}^{-1}$	[35]
(R23)	Ar(4p)→ Ar+hν	3.20 $\times \;10^{7}$	s${}^{-1}$	[35]
(R24)	Ar(4p)→ Ar${}^{m}$+hν	3.00 $\times \;10^{7}$	s${}^{-1}$	[35]
(R25)	Ar(4p)→ Ar${}^{r}$+hν	3.00 $\times \;10^{7}$	s${}^{-1}$	[35]
(R26)	Ar${}^{m}$+Ar${}^{m}$→ Ar+Ar	2.00 $\times \;10^{-13}$	m${}^{3}$s${}^{-1}$	[35]
(R27)	Ar${}^{m}$+Ar→ Ar+Ar	2.10 $\times \;10^{-21}$	m${}^{3}$s${}^{-1}$	[35]

**Table A2:** Argon-C${}_{x}$F${}_{y}$ reaction set included in a volume-averaged plasma model.

	Reaction	Rate Constant	Unit	Reference
(R28)	Ar${}^{m}$+CF${}_{2}$→ Ar+CF+F	6.00 $\times \;10^{-17}$	m${}^{3}$s${}^{-1}$	[36]
(R29)	Ar${}^{r}$+CF${}_{2}$→ Ar+CF+F	6.00 $\times \;10^{-17}$	m${}^{3}$s${}^{-1}$	[36]
(R30)	Ar${}^{m}$+CF${}_{3}$→ Ar+CF${}_{2}$+F	6.00 $\times \;10^{-17}$	m${}^{3}$s${}^{-1}$	[36]
(R31)	Ar${}^{r}$+CF${}_{3}$→ Ar+CF${}_{2}$+F	6.00 $\times \;10^{-17}$	m${}^{3}$s${}^{-1}$	[36]
(R32)	Ar${}^{m}$+CF${}_{4}$→ Ar+CF${}_{2}$+2F	6.00 $\times \;10^{-17}$	m${}^{3}$s${}^{-1}$	[36]
(R33)	Ar${}^{r}$+CF${}_{4}$→ Ar+CF${}_{2}$+2F	6.00 $\times \;10^{-17}$	m${}^{3}$s${}^{-1}$	[36]
(R34)	Ar${}^{m}$+C${}_{2}$F${}_{4}$→ Ar+2CF${}_{2}$	4.00 $\times \;10^{-17}$	m${}^{3}$s${}^{-1}$	[36]
(R35)	Ar${}^{r}$+C${}_{2}$F${}_{4}$→ Ar+2CF${}_{2}$	4.00 $\times \;10^{-17}$	m${}^{3}$s${}^{-1}$	[36]
(R36)	Ar${}^{m}$+C${}_{2}$F${}_{5}$→ Ar+C${}_{2}$F${}_{5}$	4.00 $\times \;10^{-17}$	m${}^{3}$s${}^{-1}$	[36]
(R37)	Ar${}^{r}$+C${}_{2}$F${}_{5}$→ Ar+C${}_{2}$F${}_{5}$	4.00 $\times \;10^{-17}$	m${}^{3}$s${}^{-1}$	[36]
(R38)	Ar${}^{m}$+C${}_{2}$F${}_{6}$→ Ar+2CF${}_{3}$	4.00 $\times \;10^{-17}$	m${}^{3}$s${}^{-1}$	[36]
(R39)	Ar${}^{r}$+C${}_{2}$F${}_{6}$→ Ar+2CF${}_{3}$	4.00 $\times \;10^{-17}$	m${}^{3}$s${}^{-1}$	[36]
(R40)	Ar${}^{m}$+C${}_{3}$F${}_{5}$→ Ar+C${}_{3}$F${}_{5}$	4.00 $\times \;10^{-17}$	m${}^{3}$s${}^{-1}$	[36]
(R41)	Ar${}^{r}$+C${}_{3}$F${}_{5}$→ Ar+C${}_{3}$F${}_{5}$	4.00 $\times \;10^{-17}$	m${}^{3}$s${}^{-1}$	[36]
(R42)	Ar${}^{m}$+C${}_{3}$F${}_{6}$→ Ar+C${}_{3}$F${}_{6}$	4.00 $\times \;10^{-17}$	m${}^{3}$s${}^{-1}$	[36]
(R43)	Ar${}^{r}$+C${}_{3}$F${}_{6}$→ Ar+C${}_{3}$F${}_{6}$	4.00 $\times \;10^{-17}$	m${}^{3}$s${}^{-1}$	[36]
(R44)	Ar${}^{m}$+C${}_{3}$F${}_{7}$→ Ar+C${}_{3}$F${}_{7}$	4.00 $\times \;10^{-17}$	m${}^{3}$s${}^{-1}$	[36]
(R45)	Ar${}^{r}$+C${}_{3}$F${}_{7}$→ Ar+C${}_{3}$F${}_{7}$	4.00 $\times \;10^{-17}$	m${}^{3}$s${}^{-1}$	[36]
(R46)	Ar${}^{m}$+C${}_{4}$F${}_{8}$→ Ar+2C${}_{2}$F${}_{4}$	9.00 $\times \;10^{-16}$	m${}^{3}$s${}^{-1}$	[36]
(R47)	Ar${}^{r}$+C${}_{4}$F${}_{8}$→ Ar+2C${}_{2}$F${}_{4}$	9.00 $\times \;10^{-16}$	m${}^{3}$s${}^{-1}$	[36]
(R48)	Ar${}^{+}$+CF${}_{2}$→ Ar+CF${}^{+}$+F	5.00 $\times \;10^{-16}$	m${}^{3}$s${}^{-1}$	[36]
(R49)	Ar${}^{+}$+CF${}_{3}$→ Ar+CF${}_{2}^{+}$+F	5.00 $\times \;10^{-16}$	m${}^{3}$s${}^{-1}$	[36]
(R50)	Ar${}^{+}$+CF${}_{4}$→ Ar+CF${}_{3}^{+}$+F	4.80 $\times \;10^{-16}$	m${}^{3}$s${}^{-1}$	[36]
(R51)	Ar${}^{+}$+C${}_{2}$F${}_{6}$→ Ar+CF${}_{3}^{+}$+CF${}_{3}$	5.00 $\times \;10^{-16}$	m${}^{3}$s${}^{-1}$	[36]
(R52)	Ar${}^{+}$+C${}_{4}$F${}_{8}$→ Ar+CF${}_{3}^{+}$+C${}_{3}$F${}_{5}$	3.00 $\times \;10^{-16}$	m${}^{3}$s${}^{-1}$	[36]
(R53)	Ar${}^{+}$+C${}_{4}$F${}_{8}$→ Ar+C${}_{2}$F${}_{4}^{+}$+C${}_{2}$F${}_{4}$	3.00 $\times \;10^{-16}$	m${}^{3}$s${}^{-1}$	[36]
(R54)	Ar${}^{+}$+C${}_{4}$F${}_{8}$→ Ar+CF${}^{+}$+C${}_{3}$F${}_{7}$	1.00 $\times \;10^{-16}$	m${}^{3}$s${}^{-1}$	[36]
(R55)	Ar${}^{+}$+CF${}_{3}^{-}$→ Ar+CF${}_{3}$	2.00 $\times \;10^{-13}$	m${}^{3}$s${}^{-1}$	[36]
(R56)	Ar${}^{+}$+C${}_{4}$F${}_{8}^{-}$→ Ar+C${}_{4}$F${}_{8}$	9.00 $\times \;10^{-14}$	m${}^{3}$s${}^{-1}$	[36]
(R57)	Ar${}^{+}$+F${}^{-}$→ Ar+F	2.00 $\times \;10^{-13}$	m${}^{3}$s${}^{-1}$	[36]

**Table A3:** C${}_{x}$F${}_{y}$ electron impact dissociation reaction set included in a volume-averaged plasma model.

	Reaction	Rate Constant	Unit	Reference
(R58)	e+C${}_{4}$F${}_{8}$→ e+2C${}_{2}$F${}_{4}$	0.9$\times 9.580\times 10^{-14}$T${}_{e}^{0.04153}\exp\left(-8.572/T_{e}\right)$	m${}^{3}$s${}^{-1}$	[34]
(R59)	e+C${}_{4}$F${}_{8}$→ e+C${}_{3}$F${}_{6}$+CF${}_{2}$	0.1$\times 9.580\times 10^{-14}$T${}_{e}^{0.04153}\exp\left(-8.572/T_{e}\right)$	m${}^{3}$s${}^{-1}$	[34]
(R60)	e+C${}_{4}$F${}_{7}$→ e+C${}_{2}$F${}_{4}$+C${}_{2}$F${}_{3}$	5.70 $\times \;10^{-14}$T${}_{e}^{0.28}\exp\left(-8.0/T_{e}\right)$	m${}^{3}$s${}^{-1}$	[34]
(R61)	e+C${}_{3}$F${}_{7}$→ e+C${}_{2}$F${}_{4}$+CF${}_{3}$	1.8 $\times \;10^{-14}$T${}_{e}^{0.52}\exp\left(-12.3/T_{e}\right)$	m${}^{3}$s${}^{-1}$	[34]
(R62)	e+C${}_{3}$F${}_{6}$→ e+C${}_{2}$F${}_{3}$+CF${}_{3}$	1.8 $\times \;10^{-14}$T${}_{e}^{0.52}\exp\left(-12.3/T_{e}\right)$	m${}^{3}$s${}^{-1}$	[34]
(R63)	e+C${}_{3}$F${}_{6}$→ e+C${}_{2}$F${}_{4}$+CF${}_{2}$	1.07 $\times \;10^{-14}$T${}_{e}^{0.23}\exp\left(-7.451/T_{e}\right)$	m${}^{3}$s${}^{-1}$	[34]
(R64)	e+C${}_{3}$F${}_{5}$→ e+C${}_{2}$F${}_{3}$+CF${}_{2}$	1.707 $\times \;10^{-14}$T${}_{e}^{0.23}\exp\left(-7.451/T_{e}\right)$	m${}^{3}$s${}^{-1}$	[34]
(R65)	e+C${}_{3}$F${}_{5}$→ e+C${}_{2}$F${}_{4}$+CF	1.8 $\times \;10^{-14}$T${}_{e}^{0.52}\exp\left(-12.3/T_{e}\right)$	m${}^{3}$s${}^{-1}$	[34]
(R66)	e+C${}_{2}$F${}_{6}$→ e+2CF${}_{3}$	8.55 $\times \;10^{-14}$T${}_{e}^{0.45}\exp\left(-19.4/T_{e}\right)$	m${}^{3}$s${}^{-1}$	[34]
(R67)	e+C${}_{2}$F${}_{5}$→ e+CF${}_{3}$+CF${}_{2}$	8.55 $\times \;10^{-14}$T${}_{e}^{0.45}\exp\left(-19.4/T_{e}\right)$	m${}^{3}$s${}^{-1}$	[34]
(R68)	e+C${}_{2}$F${}_{4}$→ e+2CF${}_{2}$	5.22 $\times \;10^{-12}$T${}_{e}^{-1.3815}\exp\left(-14.27/T_{e}\right)$	m${}^{3}$s${}^{-1}$	[34]
(R69)	e+C${}_{2}$F${}_{3}$→ e+CF${}_{2}$+CF	1.00 $\times \;10^{-14}$T${}_{e}^{0.91}\exp\left(-5.0/T_{e}\right)$	m${}^{3}$s${}^{-1}$	[34]
(R70)	e+CF${}_{4}$→ e+CF+F${}_{2}$+F	4.40 $\times \;10^{-16}$T${}_{e}^{0.84}\exp\left(-24.65/T_{e}\right)$	m${}^{3}$s${}^{-1}$	[34]
(R71)	e+CF${}_{4}$→ e+CF${}_{3}$+F	1.369 $\times \;10^{-16}$T${}_{e}^{2.048}\exp\left(-7.557/T_{e}\right)$	m${}^{3}$s${}^{-1}$	[34]
(R72)	e+CF${}_{4}$→ e+CF${}_{2}$+2F	1.359 $\times \;10^{-18}$T${}_{e}^{1.693}\exp\left(-13.104/T_{e}\right)$	m${}^{3}$s${}^{-1}$	[33]
(R73)	e+CF${}_{4}$→ e+CF+3F	8.215 $\times \;10^{-17}$T${}_{e}^{0.277}\exp\left(-27.151/T_{e}\right)$	m${}^{3}$s${}^{-1}$	[33]
(R74)	e+CF${}_{3}$→ e+CF${}_{2}$+F	3.257 $\times \;10^{-15}$T${}_{e}^{0.6906}\exp\left(-3.65/T_{e}\right)$	m${}^{3}$s${}^{-1}$	[33]
(R75)	e+CF${}_{2}$→ e+CF+F	3.257 $\times \;10^{-15}$T${}_{e}^{0.6906}\exp\left(-5.39/T_{e}\right)$	m${}^{3}$s${}^{-1}$	[33]
(R76)	e+CF${}_{2}$(s)→ e+CF+F	19.12 $\times \;10^{-16}$T${}_{e}^{-1.496}\exp\left(-7.238/T_{e}\right)$	m${}^{3}$s${}^{-1}$	[33]
(R77)	e+CF${}_{2}$→ e+C+2F	1.39 $\times \;10^{-14}$T${}_{e}^{-1.164}\exp\left(-49.873/T_{e}\right)$	m${}^{3}$s${}^{-1}$	[34]
(R78)	e+CF→ e+C+F	5.633 $\times \;10^{-14}$T${}_{e}^{-1.318}\exp\left(-7.158/T_{e}\right)$	m${}^{3}$s${}^{-1}$	[33]
(R79)	e+CF(s)→ e+C+F	5.633 $\times \;10^{-14}$T${}_{e}^{-1.318}\exp\left(-4.248/T_{e}\right)$	m${}^{3}$s${}^{-1}$	[33]
(R80)	e+F${}_{2}$→ e+F+F	4.983 $\times \;10^{-15}$T${}_{e}^{0.4}\exp\left(-3.947/T_{e}\right)$	m${}^{3}$s${}^{-1}$	[33]
(R81)	e+F${}_{2}^{-}$→ e+F+F${}^{-}$	5.812 $\times \;10^{-15}$T${}_{e}^{0.062}\exp\left(-11.498/T_{e}\right)$	m${}^{3}$s${}^{-1}$	[33]

**Table A4:** C${}_{x}$F${}_{y}$ electron impact ionization reaction set included in a volume-averaged plasma model.

	Reaction	Rate Constant	Unit	Reference
(R82)	e+C${}_{4}$F${}_{8}$→ 2e+C${}_{3}$F${}_{5}^{+}$+CF${}_{3}$	6.655 $\times \;10^{-14}$T${}_{e}^{0.4095}\exp\left(-18.71/T_{e}\right)$	m${}^{3}$s${}^{-1}$	[34]
(R83)	e+C${}_{4}$F${}_{8}$→ 2e+C${}_{3}$F${}_{5}^{+}$+CF${}_{2}$+F	6.655 $\times \;10^{-14}$T${}_{e}^{0.4095}\exp\left(-18.71/T_{e}\right)$	m${}^{3}$s${}^{-1}$	[34]
(R84)	e+C${}_{4}$F${}_{8}$→ 2e+C${}_{2}$F${}_{4}^{+}$+C${}_{2}$F${}_{4}$	5.698 $\times \;10^{-14}$T${}_{e}^{0.470}\exp\left(-17.48/T_{e}\right)$	m${}^{3}$s${}^{-1}$	[34]
(R85)	e+C${}_{4}$F${}_{8}$→ 2e+CF${}_{3}^{+}$+C${}_{3}$F${}_{5}$	2.688 $\times \;10^{-14}$T${}_{e}^{0.379}\exp\left(-22.30/T_{e}\right)$	m${}^{3}$s${}^{-1}$	[34]
(R86)	e+C${}_{4}$F${}_{8}$→ 2e+CF${}_{2}^{+}$+C${}_{3}$F${}_{6}$	4.840 $\times \;10^{-14}$T${}_{e}^{-0.02637}\exp\left(-27.03/T_{e}\right)$	m${}^{3}$s${}^{-1}$	[34]
(R87)	e+C${}_{4}$F${}_{8}$→ 2e+CF${}^{+}$+C${}_{3}$F${}_{7}$	0.5 $\times \;1.279\times 10^{-14}$T${}_{e}^{0.654}\exp\left(-20.61/T_{e}\right)$	m${}^{3}$s${}^{-1}$	[34]
(R88)	e+C${}_{4}$F${}_{8}$→ 2e+CF${}^{+}$+C${}_{3}$F${}_{6}$+F	0.5 $\times \;1.279\times 10^{-14}$T${}_{e}^{0.654}\exp\left(-20.61/T_{e}\right)$	m${}^{3}$s${}^{-1}$	[34]
(R89)	e+C${}_{4}$F${}_{8}$→ 2e+F${}^{+}$+C${}_{4}$F${}_{7}$	2.23 $\times \;1.279\times 10^{-14}$T${}_{e}^{-1.34}\exp\left(-38.6/T_{e}\right)$	m${}^{3}$s${}^{-1}$	[34]
(R90)	e+C${}_{4}$F${}_{7}$→ 2e+C${}_{4}$F${}_{7}^{+}$	1.4 $\times \;10^{-14}$T${}_{e}^{0.68}\exp\left(-10.6/T_{e}\right)$	m${}^{3}$s${}^{-1}$	[34]
(R91)	e+C${}_{3}$F${}_{6}$→ 2e+C${}_{3}$F${}_{6}^{+}$	1.4 $\times \;10^{-14}$T${}_{e}^{0.68}\exp\left(-10.6/T_{e}\right)$	m${}^{3}$s${}^{-1}$	[34]
(R92)	e+C${}_{3}$F${}_{6}$→ 2e+C${}_{3}$F${}_{5}^{+}$+F	3.025 $\times \;10^{-15}$T${}_{e}^{0.874}\exp\left(-16.41/T_{e}\right)$	m${}^{3}$s${}^{-1}$	[34]
(R93)	e+C${}_{3}$F${}_{5}$→ 2e+C${}_{3}$F${}_{6}^{+}$	3.583 $\times \;10^{-15}$T${}_{e}^{0.661}\exp\left(-11.06/T_{e}\right)$	m${}^{3}$s${}^{-1}$	[34]
(R94)	e+C${}_{2}$F${}_{6}$→ 2e+CF${}_{3}^{+}$+CF${}_{3}$	4.83 $\times \;10^{-14}$T${}_{e}^{0.61}\exp\left(-19.47/T_{e}\right)$	m${}^{3}$s${}^{-1}$	[34]
(R95)	e+C${}_{2}$F${}_{5}$→ 2e+C${}_{2}$F${}_{5}^{+}$	1.435 $\times \;10^{-14}$T${}_{e}^{0.39}\exp\left(-15.38/T_{e}\right)$	m${}^{3}$s${}^{-1}$	[34]
(R96)	e+C${}_{2}$F${}_{5}$→ 2e+CF${}_{3}^{+}$+CF${}_{2}$	6.01 $\times \;10^{-14}$T${}_{e}^{0.30}\exp\left(-19.15/T_{e}\right)$	m${}^{3}$s${}^{-1}$	[34]
(R97)	e+C${}_{2}$F${}_{4}$→ 2e+C${}_{2}$F${}_{4}^{+}$	3.583 $\times \;10^{-15}$T${}_{e}^{0.661}\exp\left(-11.06/T_{e}\right)$	m${}^{3}$s${}^{-1}$	[34]
(R98)	e+C${}_{2}$F${}_{4}$→ 2e+C${}_{2}$F${}_{3}^{+}$+F	3.025 $\times \;10^{-15}$T${}_{e}^{0.874}\exp\left(-16.41/T_{e}\right)$	m${}^{3}$s${}^{-1}$	[34]
(R99)	e+C${}_{2}$F${}_{4}$→ 2e+CF${}_{2}^{+}$+CF${}_{2}$	1.253 $\times \;10^{-16}$T${}_{e}^{1.514}\exp\left(-9.053/T_{e}\right)$	m${}^{3}$s${}^{-1}$	[34]
(R100)	e+C${}_{2}$F${}_{4}$→ 2e+CF${}^{+}$+CF${}_{3}$	5.874 $\times \;10^{-15}$T${}_{e}^{0.619}\exp\left(-19.29/T_{e}\right)$	m${}^{3}$s${}^{-1}$	[34]
(R101)	e+C${}_{2}$F${}_{3}$→ 2e+C${}_{2}$F${}_{3}^{+}$	3.583 $\times \;10^{-15}$T${}_{e}^{0.661}\exp\left(-11.06/T_{e}\right)$	m${}^{3}$s${}^{-1}$	[34]
(R102)	e+CF${}_{4}$→ 2e+CF${}_{3}^{+}$+F	1.083 $\times \;10^{-14}$T${}_{e}^{0.969}\exp\left(-17.803/T_{e}\right)$	m${}^{3}$s${}^{-1}$	[33]
(R103)	e+CF${}_{4}$→ 2e+CF${}_{2}^{+}$+2F	3.310 $\times \;10^{-16}$T${}_{e}^{1.365}\exp\left(-18.373/T_{e}\right)$	m${}^{3}$s${}^{-1}$	[33]
(R104)	e+CF${}_{4}$→ 2e+CF${}^{+}$+3F	7.171 $\times \;10^{-19}$T${}_{e}^{3.453}\exp\left(-14.244/T_{e}\right)$	m${}^{3}$s${}^{-1}$	[33]
(R105)	e+CF${}_{4}$→ 2e+F${}^{+}$+CF3	1.297 $\times \;10^{-18}$T${}_{e}^{2.786}\exp\left(-18.557/T_{e}\right)$	m${}^{3}$s${}^{-1}$	[33]
(R106)	e+CF${}_{4}$→ 2e+C${}^{+}$+4F	9.154 $\times \;10^{-20}$T${}_{e}^{3.847}\exp\left(-12.082/T_{e}\right)$	m${}^{3}$s${}^{-1}$	[33]
(R107)	e+CF${}_{4}$→ 3e+CF${}_{3}^{+}$+F${}^{+}$	5.611 $\times \;10^{-17}$T${}_{e}^{1.157}\exp\left(-37.455/T_{e}\right)$	m${}^{3}$s${}^{-1}$	[33]
(R108)	e+CF${}_{4}$→ 3e+CF${}_{2}^{+}$+F${}^{+}$+F	8.207 $\times \;10^{-17}$T${}_{e}^{1.163}\exp\left(-42.737/T_{e}\right)$	m${}^{3}$s${}^{-1}$	[33]
(R109)	e+CF${}_{4}$→ 3e+CF${}^{+}$+F${}^{+}$+2F	5.217 $\times \;10^{-17}$T${}_{e}^{1.52}\exp\left(-47.145/T_{e}\right)$	m${}^{3}$s${}^{-1}$	[33]
(R110)	e+CF${}_{4}$→ 3e+C${}^{+}$+F${}^{+}$+3F	4.075 $\times \;10^{-17}$T${}_{e}^{1.353}\exp\left(-61.618/T_{e}\right)$	m${}^{3}$s${}^{-1}$	[33]
(R111)	e+CF${}_{3}$→ 2e+CF${}_{3}^{+}$	1.427 $\times \;10^{-15}$T${}_{e}^{0.838}\exp\left(-9.549/T_{e}\right)$	m${}^{3}$s${}^{-1}$	[33]
(R112)	e+CF${}_{3}$→ 2e+CF${}_{2}^{+}$+F	8.362 $\times \;10^{-15}$T${}_{e}^{0.444}\exp\left(-17.368/T_{e}\right)$	m${}^{3}$s${}^{-1}$	[33]
(R113)	e+CF${}_{3}$→ 2e+CF${}^{+}$+2F	5.257 $\times \;10^{-15}$T${}_{e}^{0.555}\exp\left(-21.232/T_{e}\right)$	m${}^{3}$s${}^{-1}$	[33]
(R114)	e+CF${}_{3}$→ 2e+F${}^{+}$+CF${}_{2}$	2.669 $\times \;10^{-15}$T${}_{e}^{0.820}\exp\left(-27.018/T_{e}\right)$	m${}^{3}$s${}^{-1}$	[33]
(R115)	e+CF${}_{2}$→ 2e+CF${}_{2}^{+}$	4.330 $\times \;10^{-15}$T${}_{e}^{0.775}\exp\left(-9.485/T_{e}\right)$	m${}^{3}$s${}^{-1}$	[33]
(R116)	e+CF${}_{2}$→ 2e+CF${}^{+}$+F	6.032 $\times \;10^{-15}$T${}_{e}^{0.497}\exp\left(-14.539/T_{e}\right)$	m${}^{3}$s${}^{-1}$	[33]
(R117)	e+CF${}_{2}$→ 3e+CF${}^{+}$+F${}^{+}$	3.176 $\times \;10^{-14}$T${}_{e}^{-0.111}\exp\left(-31.314/T_{e}\right)$	m${}^{3}$s${}^{-1}$	[33]
(R118)	e+CF${}_{2}$→ 2e+CF+F${}^{+}$	1.588 $\times \;10^{-14}$T${}_{e}^{-0.111}\exp\left(-31.314/T_{e}\right)$	m${}^{3}$s${}^{-1}$	[33]
(R119)	e+CF→ 2e+CF${}^{+}$	4.295 $\times \;10^{-15}$T${}_{e}^{-0.80}\exp\left(-11.541/T_{e}\right)$	m${}^{3}$s${}^{-1}$	[33]
(R120)	e+CF→ 2e+C${}^{+}$+F${}^{+}$	1.985 $\times \;10^{-14}$T${}_{e}^{-0.111}\exp\left(-31.314/T_{e}\right)$	m${}^{3}$s${}^{-1}$	[33]
(R121)	e+F${}_{2}$→ 2e+F${}_{2}^{+}$	6.710 $\times \;10^{-16}$T${}_{e}^{1.24}\exp\left(-16.822/T_{e}\right)$	m${}^{3}$s${}^{-1}$	[33]
(R122)	e+C→ 2e+C${}^{+}$	3.692 $\times \;10^{-15}$T${}_{e}^{1.182}\exp\left(-9.332/T_{e}\right)$	m${}^{3}$s${}^{-1}$	[33]
(R123)	e+CF${}_{2}{(}^{3}B_{1})$→ 2e+CF${}_{2}^{+}$	4.330 $\times \;10^{-15}$T${}_{e}^{0.775}\exp\left(-7.235/T_{e}\right)$	m${}^{3}$s${}^{-1}$	[33]
(R124)	e+CF${}_{2}{(}^{3}B_{1})$→ 2e+CF${}^{+}$+F	6.032 $\times \;10^{-15}$T${}_{e}^{0.497}\exp\left(-12.289/T_{e}\right)$	m${}^{3}$s${}^{-1}$	[33]
(R125)	e+CF${}_{2}{(}^{3}B_{1})$→ 3e+CF${}^{+}$+F${}^{+}$	3.176 $\times \;10^{-14}$T${}_{e}^{-0.111}\exp\left(-29.064/T_{e}\right)$	m${}^{3}$s${}^{-1}$	[33]
(R126)	e+CF${}_{2}{(}^{3}B_{1})$→ 2e+CF+F${}^{+}$	1.588 $\times \;10^{-14}$T${}_{e}^{-0.111}\exp\left(-29.064/T_{e}\right)$	m${}^{3}$s${}^{-1}$	[33]
(R127)	e+CF$\left(a^{4}\Sigma^{-}\right)$→ 2e+CF${}^{+}$	4.295 $\times \;10^{-15}$T${}_{e}^{0.8}\exp\left(-8.631/T_{e}\right)$	m${}^{3}$s${}^{-1}$	[33]
(R128)	e+CF$\left(a^{4}\Sigma^{-}\right)$→ 2e+C+F${}^{+}$	1.985 $\times \;10^{-14}$T${}_{e}^{-0.111}\exp\left(-28.404/T_{e}\right)$	m${}^{3}$s${}^{-1}$	[33]
(R129)	e+F${}_{2}$→ e+F${}^{+}$+F${}^{-}$	9.432 $\times \;10^{-17}$T${}_{e}^{1.341}\exp\left(-17.322/T_{e}\right)$	m${}^{3}$s${}^{-1}$	[33]
(R130)	e+F→ e+F${}^{+}$	4.711 $\times \;10^{-16}$T${}_{e}^{1.411}\exp\left(-12.618/T_{e}\right)$	m${}^{3}$s${}^{-1}$	[33]

**Table A5:** C${}_{x}$F${}_{y}$ attachment and detachment reaction sets included in a volume-averaged plasma model.

	Reaction	Rate Constant	Unit	Reference
(R131)	e+C${}_{4}$F${}_{8}$→ C${}_{4}$F${}_{8}^{-}$	2.960 $\times \;10^{-17}$T${}_{e}^{-1.328}\exp\left(-0.2344/T_{e}\right)$	m${}^{3}$s${}^{-1}$	[34]
(R132)	e+C${}_{4}$F${}_{8}$→ F${}^{-}$+C${}_{4}$F${}_{7}$	0.5$\times 2.789\times 10^{-15}$T${}_{e}^{-1.277}\exp\left(-5.392/T_{e}\right)$	m${}^{3}$s${}^{-1}$	[34]
(R133)	e+C${}_{4}$F${}_{8}$→ F${}^{-}$+C${}_{3}$F${}_{6}$+F	0.5$\times 2.789\times 10^{-15}$T${}_{e}^{-1.277}\exp\left(-5.392/T_{e}\right)$	m${}^{3}$s${}^{-1}$	[34]
(R134)	e+C${}_{2}$F${}_{6}$→ CF${}_{3}^{-}$+CF${}_{3}$	0.8$\times 6.82\times 10^{-15}$T${}_{e}^{-1.41}\exp\left(-4.05/T_{e}\right)$	m${}^{3}$s${}^{-1}$	[34]
(R135)	e+C${}_{2}$F${}_{6}$→ CF${}^{-}$+C${}_{2}$F${}_{5}$	0.2$\times 6.82\times 10^{-15}$T${}_{e}^{-1.41}\exp\left(-4.05/T_{e}\right)$	m${}^{3}$s${}^{-1}$	[34]
(R136)	e+C${}_{2}$F${}_{5}$→ CF${}_{3}^{-}$+CF${}_{2}$	6.82 $\times \;10^{-15}$T${}_{e}^{-1.41}\exp\left(-4.05/T_{e}\right)$	m${}^{3}$s${}^{-1}$	[34]
(R137)	e+CF${}_{4}$→ CF${}_{3}$+F${}^{-}$	1.086 $\times \;10^{-15}$T${}_{e}^{-1.336}\exp\left(-6.691/T_{e}\right)$	m${}^{3}$s${}^{-1}$	[33]
(R138)	e+CF${}_{4}$→ CF${}_{2}$+F${}_{2}^{-}$	1.960 $\times \;10^{-17}$T${}_{e}^{-1.300}\exp\left(-6.247/T_{e}\right)$	m${}^{3}$s${}^{-1}$	[33]
(R139)	e+CF${}_{4}$→ CF${}_{3}^{-}$+F	2.545 $\times \;10^{-16}$T${}_{e}^{-1.374}\exp\left(-7.12/T_{e}\right)$	m${}^{3}$s${}^{-1}$	[33]
(R140)	e+CF${}_{4}$→ e+CF${}_{3}^{+}$+F${}^{-}$	4.086 $\times \;10^{-19}$T${}_{e}^{0.65}\exp\left(-16.738/T_{e}\right)$	m${}^{3}$s${}^{-1}$	[33]
(R141)	e+CF${}_{4}$→ e+CF${}_{2}^{+}$+F${}^{-}$+F	2.567 $\times \;10^{-19}$T${}_{e}^{0.412}\exp\left(-21.866/T_{e}\right)$	m${}^{3}$s${}^{-1}$	[33]
(R142)	e+CF${}_{4}$→ e+CF${}^{+}$+F${}^{-}$+2F	8.439 $\times \;10^{-19}$T${}_{e}^{0.723}\exp\left(-21.994/T_{e}\right)$	m${}^{3}$s${}^{-1}$	[33]
(R143)	e+CF${}_{4}$→ e+F${}^{+}$+F${}^{-}$+CF${}_{2}$	5.087 $\times \;10^{-19}$T${}_{e}^{0.636}\exp\left(-27.714/T_{e}\right)$	m${}^{3}$s${}^{-1}$	[33]
(R144)	e+CF${}_{4}$→ e+C${}^{+}$+F${}^{-}$+3F	2.395 $\times \;10^{-18}$T${}_{e}^{0.42}\exp\left(-28.416/T_{e}\right)$	m${}^{3}$s${}^{-1}$	[33]
(R145)	e+CF${}_{4}$→ e+CF${}_{2}^{+}$+F${}_{2}^{-}$	3.691 $\times \;10^{-19}$T${}_{e}^{0.051}\exp\left(-10.163/T_{e}\right)$	m${}^{3}$s${}^{-1}$	[33]
(R146)	e+CF${}_{3}$→ e+CF${}_{2}$+F${}^{-}$	1.603 $\times \;10^{-16}$T${}_{e}^{-0.4289}\exp\left(-0.18/T_{e}\right)$	m${}^{3}$s${}^{-1}$	[44]
(R147)	e+CF${}_{2}$→ F${}^{-}$+CF	3.0 $\times \;10^{-17}$	m${}^{3}$s${}^{-1}$	[34]
(R148)	e+CF→ F${}^{-}$+C	3.0 $\times \;10^{-17}$	m${}^{3}$s${}^{-1}$	[34]
(R149)	e+F${}_{2}^{-}$→ 2e+F${}_{2}$	6.186 $\times \;10^{-16}$T${}_{e}^{-0.535}\exp\left(-11.092/T_{e}\right)$	m${}^{3}$s${}^{-1}$	[44]
(R150)	e+F${}_{2}$→ F+F${}^{-}$	1.124 $\times \;10^{-15}$T${}_{e}^{-1.475}\exp\left(-0.535/T_{e}\right)$	m${}^{3}$s${}^{-1}$	[33]
(R151)	e+F${}^{-}$→ 2e+F	3.047 $\times \;10^{-14}$T${}_{e}^{0.413}\exp\left(-11.167/T_{e}\right)$	m${}^{3}$s${}^{-1}$	[44]
(R152)	C${}_{4}$F${}_{8}^{-}$→ e+C${}_{4}$F${}_{8}$	2.0 $\times \;10^{-6}$	s${}^{-1}$	[34]
(R153)	CF${}_{3}^{-}$+CF${}_{3}$→ e+C${}_{2}$F${}_{6}$	1.0 $\times \;10^{-16}$	m${}^{3}$s${}^{-1}$	[34]
(R154)	F${}^{-}$+CF${}_{4}$→ e+CF${}_{4}$	2.132 $\times \;10^{-14}\times {T_{i}}^{1.131}\exp\left(-1.448/T_{i}\right)$	m${}^{3}$s${}^{-1}$	[33]
(R155)	F${}^{-}$+CF${}_{3}$→ e+CF${}_{4}$	5.0 $\times \;10^{-16}$	m${}^{3}$s${}^{-1}$	[33]
(R156)	F${}^{-}$+CF${}_{2}$→ e+CF${}_{3}$	5.0 $\times \;10^{-16}$	m${}^{3}$s${}^{-1}$	[33]
(R157)	F${}^{-}$+CF→ e+CF${}_{2}$	5.0 $\times \;10^{-16}$	m${}^{3}$s${}^{-1}$	[33]
(R158)	F${}^{-}$+C→ e+CF	1.0 $\times \;10^{-16}$	m${}^{3}$s${}^{-1}$	[33]
(R159)	F${}^{-}$+F→ e+F${}_{2}$	1.0 $\times \;10^{-16}$	m${}^{3}$s${}^{-1}$	[33]
(R160)	F${}^{-}$+F→ e+2F	1.0 $\times \;10^{-16}$	m${}^{3}$s${}^{-1}$	[33]
(R161)	F${}_{2}^{-}$+CF${}_{4}$→ e+F${}_{2}$+CF${}_{4}$	5.0 $\times \;10^{-16}$	m${}^{3}$s${}^{-1}$	[33]
(R162)	F${}_{2}^{-}$+CF${}_{3}$→ e+F${}_{2}$+CF${}_{3}$	5.0 $\times \;10^{-16}$	m${}^{3}$s${}^{-1}$	[33]
(R163)	F${}_{2}^{-}$+F→ e+F${}_{2}$+F	1.0 $\times \;10^{-16}$	m${}^{3}$s${}^{-1}$	[33]

**Table A6:** C${}_{x}$F${}_{y}$ recombination reaction sets included in a volume-averaged plasma model.

	Reaction	Rate Constant	Unit	Reference
(R164)	e+C${}_{4}$F${}_{7}^{+}$→ C${}_{2}$F${}_{4}$+C${}_{2}$F${}_{3}$	8.0 $\times \;10^{-14}$T${}_{e}^{-0.5}$	m${}^{3}$s${}^{-1}$	[34]
(R165)	e+C${}_{3}$F${}_{7}^{+}$→ C${}_{2}$F${}_{4}$+CF${}_{3}$	8.0 $\times \;10^{-14}$T${}_{e}^{-0.5}$	m${}^{3}$s${}^{-1}$	[34]
(R166)	e+C${}_{3}$F${}_{6}^{+}$→ C${}_{2}$F${}_{4}$+CF${}_{2}$	8.0 $\times \;10^{-14}$T${}_{e}^{-0.5}$	m${}^{3}$s${}^{-1}$	[34]
(R167)	e+C${}_{3}$F${}_{5}^{+}$→ C${}_{2}$F${}_{3}$+CF${}_{2}$	8.0 $\times \;10^{-14}$T${}_{e}^{-0.5}$	m${}^{3}$s${}^{-1}$	[34]
(R168)	e+C${}_{2}$F${}_{5}^{+}$→ CF${}_{3}$+CF${}_{2}$	8.0 $\times \;10^{-14}$T${}_{e}^{-0.5}$	m${}^{3}$s${}^{-1}$	[34]
(R169)	e+C${}_{2}$F${}_{4}^{+}$→ 2CF${}_{2}$	8.0 $\times \;10^{-14}$T${}_{e}^{-0.5}$	m${}^{3}$s${}^{-1}$	[34]
(R170)	e+C${}_{2}$F${}_{3}^{+}$→ CF${}_{2}$+CF	8.0 $\times \;10^{-14}$T${}_{e}^{-0.5}$	m${}^{3}$s${}^{-1}$	[34]
(R171)	e+CF${}_{3}^{+}$→ CF${}_{3}$	3.95 $\times \;10^{-15}$T${}_{e}^{-0.5}$T${}_{i}^{-1.0}$	m${}^{3}$s${}^{-1}$	[33]
(R172)	e+CF${}_{3}^{+}$→ CF${}_{2}$+F	4.475 $\times \;10^{-15}$T${}_{e}^{-1.441}\exp\left(-0.41/T_{e}\right)$	m${}^{3}$s${}^{-1}$	[33]
(R173)	e+CF${}_{3}^{+}$→ CF+2F	1.119 $\times \;10^{-15}$T${}_{e}^{-1.441}\exp\left(-0.41/T_{e}\right)$	m${}^{3}$s${}^{-1}$	[33]
(R174)	e+CF${}_{2}^{+}$→ CF${}_{2}$	6.00 $\times \;10^{-14}$	m${}^{3}$s${}^{-1}$	[33]
(R175)	e+CF${}_{2}^{+}$→ CF+F	8.376 $\times \;10^{-15}$T${}_{e}^{-1.496}\exp\left(-0.172/T_{e}\right)$	m${}^{3}$s${}^{-1}$	[33]
(R176)	e+CF${}_{2}^{+}$→ C+2F	3.421 $\times \;10^{-15}$T${}_{e}^{-1.496}\exp\left(-0.172/T_{e}\right)$	m${}^{3}$s${}^{-1}$	[33]
(R177)	e+CF${}^{+}$→ CF	5.20 $\times \;10^{-14}$(T${}_{e}{/0.026)}^{-0.8}$	m${}^{3}$s${}^{-1}$	[33]
(R178)	e+F${}_{2}^{+}$→ 2F	3.21 $\times \;10^{-14}$T${}_{e}^{-0.5}$	m${}^{3}$s${}^{-1}$	[33]
(R179)	e+F${}_{2}^{-}$→ 2e+2F	1.354 $\times \;10^{-14}$T${}_{e}^{0.484}\exp\left(-0.2.178/T_{e}\right)$	m${}^{3}$s${}^{-1}$	[33]
(R180)	C${}_{4}$F${}_{7}$+F→ 2C${}_{2}$F${}_{4}$	1.0 $\times \;10^{-17}$	m${}^{3}$s${}^{-1}$	[34]
(R181)	C${}_{3}$F${}_{6}$+F${}_{2}$→ C${}_{3}$F${}_{7}$+F	3.5 $\times \;10^{-22}$	m${}^{3}$s${}^{-1}$	[34]
(R182)	C${}_{3}$F${}_{6}$+F→ C${}_{3}$F${}_{7}$	1.0 $\times \;10^{-18}$	m${}^{3}$s${}^{-1}$	[34]
(R183)	C${}_{2}$F${}_{5}$+F→ 2CF${}_{3}$	1.0 $\times \;10^{-17}$	m${}^{3}$s${}^{-1}$	[34]
(R184)	C${}_{2}$F${}_{4}$+F${}_{2}$→ C${}_{2}$F${}_{5}$+F	3.5 $\times \;10^{-22}$	m${}^{3}$s${}^{-1}$	[34]
(R185)	C${}_{2}$F${}_{4}$+F→ CF${}_{3}$+CF${}_{2}$	4.8 $\times \;10^{-17}$	m${}^{3}$s${}^{-1}$	[34]
(R186)	C${}_{2}$F${}_{4}$+C→ C${}_{2}$F${}_{3}$+CF	1.91 $\times \;10^{-16}$	m${}^{3}$s${}^{-1}$	[34]
(R187)	C${}_{2}$F${}_{3}$+F→ C${}_{2}$F${}_{F}$	1.0 $\times \;10^{-18}$	m${}^{3}$s${}^{-1}$	[34]
(R188)	CF${}_{3}$+CF${}_{3}$+M→ C${}_{2}$F${}_{6}$+M	3.94 $\times \;10^{-41}$	m${}^{6}$s${}^{-1}$	[34]
(R189)	CF${}_{3}$+CF${}_{3}$→ C${}_{2}$F${}_{6}$	8.30 $\times \;10^{-18}$	m${}^{3}$s${}^{-1}$	[34]
(R190)	CF${}_{3}$+CF${}_{2}$→ C${}_{2}$F${}_{5}$	1.00 $\times \;10^{-18}$	m${}^{3}$s${}^{-1}$	[34]
(R191)	CF${}_{3}$+F${}_{2}$→ CF${}_{4}$+F	1.90 $\times \;10^{-20}$	m${}^{3}$s${}^{-1}$	[34]
(R192)	CF${}_{3}$+F→ CF${}_{4}$	2.00 $\times \;10^{-17}$	m${}^{3}$s${}^{-1}$	[34]
(R193)	CF${}_{2}$+CF${}_{2}$→ C${}_{2}$F${}_{4}$	7.21 $\times \;10^{-20}$	m${}^{3}$s${}^{-1}$	[34]
(R194)	CF${}_{2}$+F${}_{2}$→ CF${}_{3}$+F	8.30 $\times \;10^{-20}$	m${}^{3}$s${}^{-1}$	[34]
(R195)	CF${}_{2}$+F→ CF${}_{3}$	1.80 $\times \;10^{-17}$	m${}^{3}$s${}^{-1}$	[34]
(R196)	CF+F→ CF${}_{2}$	9.96 $\times \;10^{-17}$	m${}^{3}$s${}^{-1}$	[34]
(R197)	C${}_{4}$F${}_{7}^{+}$+C${}_{4}$F${}_{8}^{-}$→ C${}_{4}$F${}_{7}$+C${}_{4}$F${}_{8}$	1.0 $\times \;10^{-13}$	m${}^{3}$s${}^{-1}$	[34]
(R198)	C${}_{3}$F${}_{7}^{+}$+C${}_{4}$F${}_{8}^{-}$→ C${}_{3}$F${}_{7}$+C${}_{4}$F${}_{8}$	1.0 $\times \;10^{-13}$	m${}^{3}$s${}^{-1}$	[34]
(R199)	C${}_{3}$F${}_{6}^{+}$+C${}_{4}$F${}_{8}^{-}$→ C${}_{3}$F${}_{6}$+C${}_{4}$F${}_{8}$	1.0 $\times \;10^{-13}$	m${}^{3}$s${}^{-1}$	[34]
(R200)	C${}_{3}$F${}_{5}^{+}$+C${}_{4}$F${}_{8}^{-}$→ C${}_{3}$F${}_{5}$+C${}_{4}$F${}_{8}$	1.0 $\times \;10^{-13}$	m${}^{3}$s${}^{-1}$	[34]
(R201)	C${}_{2}$F${}_{5}^{+}$+C${}_{4}$F${}_{8}^{-}$→ C${}_{2}$F${}_{5}$+C${}_{4}$F${}_{8}$	1.0 $\times \;10^{-13}$	m${}^{3}$s${}^{-1}$	[34]
(R202)	C${}_{2}$F${}_{4}^{+}$+C${}_{4}$F${}_{8}^{-}$→ C${}_{2}$F${}_{4}$+C${}_{4}$F${}_{8}$	1.0 $\times \;10^{-13}$	m${}^{3}$s${}^{-1}$	[34]
(R203)	C${}_{2}$F${}_{3}^{+}$+C${}_{4}$F${}_{8}^{-}$→ C${}_{2}$F${}_{3}$+C${}_{4}$F${}_{8}$	1.0 $\times \;10^{-13}$	m${}^{3}$s${}^{-1}$	[34]
(R204)	CF${}_{3}^{+}$+C${}_{4}$F${}_{8}^{-}$→ CF${}_{3}$+C${}_{4}$F${}_{8}$	1.0 $\times \;10^{-13}$	m${}^{3}$s${}^{-1}$	[34]
(R205)	CF${}_{2}^{+}$+C${}_{4}$F${}_{8}^{-}$→ CF${}_{2}$+C${}_{4}$F${}_{8}$	1.0 $\times \;10^{-13}$	m${}^{3}$s${}^{-1}$	[34]
(R206)	F${}_{2}^{+}$+C${}_{4}$F${}_{8}^{-}$→ F${}_{2}$+C${}_{4}$F${}_{8}$	1.0 $\times \;10^{-13}$	m${}^{3}$s${}^{-1}$	[34]
(R207)	F${}^{+}$+C${}_{4}$F${}_{8}^{-}$→ F+C${}_{4}$F${}_{8}$	1.0 $\times \;10^{-13}$	m${}^{3}$s${}^{-1}$	[34]
(R208)	C${}^{+}$+C${}_{4}$F${}_{8}^{-}$→ C+C${}_{4}$F${}_{8}$	1.0 $\times \;10^{-13}$	m${}^{3}$s${}^{-1}$	[34]
(R209)	C${}_{4}$F${}_{7}^{+}$+CF${}_{3}^{-}$→ C${}_{4}$F${}_{7}$+CF${}_{3}$	1.0 $\times \;10^{-13}$	m${}^{3}$s${}^{-1}$	[34]
(R210)	C${}_{3}$F${}_{7}^{+}$+CF${}_{3}^{-}$→ C${}_{3}$F${}_{7}$+CF${}_{3}$	1.0 $\times \;10^{-13}$	m${}^{3}$s${}^{-1}$	[34]
(R211)	C${}_{3}$F${}_{6}^{+}$+CF${}_{3}^{-}$→ C${}_{3}$F${}_{6}$+CF${}_{3}$	1.0 $\times \;10^{-13}$	m${}^{3}$s${}^{-1}$	[34]
(R212)	C${}_{3}$F${}_{6}^{+}$+CF${}_{3}^{-}$→ C${}_{3}$F${}_{6}$+CF${}_{3}$	1.0 $\times \;10^{-13}$	m${}^{3}$s${}^{-1}$	[34]
(R213)	C${}_{2}$F${}_{5}^{+}$+CF${}_{3}^{-}$→ C${}_{2}$F${}_{5}$+CF${}_{3}$	1.0 $\times \;10^{-13}$	m${}^{3}$s${}^{-1}$	[34]
(R214)	C${}_{2}$F${}_{4}^{+}$+CF${}_{3}^{-}$→ C${}_{2}$F${}_{4}$+CF${}_{3}$	1.0 $\times \;10^{-13}$	m${}^{3}$s${}^{-1}$	[34]
(R215)	C${}_{2}$F${}_{3}^{+}$+CF${}_{3}^{-}$→ C${}_{2}$F${}_{3}$+CF${}_{3}$	1.0 $\times \;10^{-13}$	m${}^{3}$s${}^{-1}$	[34]
(R216)	C${}_{4}$F${}_{7}^{+}$+F${}_{2}^{-}$→ C${}_{4}$F${}_{7}$+F${}_{2}$	1.0 $\times \;10^{-13}$	m${}^{3}$s${}^{-1}$	[34]
(R217)	C${}_{3}$F${}_{7}^{+}$+F${}_{2}^{-}$→ C${}_{3}$F${}_{7}$+F${}_{2}$	1.0 $\times \;10^{-13}$	m${}^{3}$s${}^{-1}$	[34]
(R218)	C${}_{3}$F${}_{6}^{+}$+F${}_{2}^{-}$→ C${}_{3}$F${}_{6}$+F${}_{2}$	1.0 $\times \;10^{-13}$	m${}^{3}$s${}^{-1}$	[34]
(R219)	C${}_{3}$F${}_{5}^{+}$+F${}_{2}^{-}$→ C${}_{3}$F${}_{5}$+F${}_{2}$	1.0 $\times \;10^{-13}$	m${}^{3}$s${}^{-1}$	[34]
(R220)	C${}_{2}$F${}_{5}^{+}$+F${}_{2}^{-}$→ C${}_{2}$F${}_{5}$+F${}_{2}$	1.0 $\times \;10^{-13}$	m${}^{3}$s${}^{-1}$	[34]
(R221)	C${}_{2}$F${}_{4}^{+}$+F${}_{2}^{-}$→ C${}_{2}$F${}_{4}$+F${}_{2}$	1.0 $\times \;10^{-13}$	m${}^{3}$s${}^{-1}$	[34]
(R222)	C${}_{2}$F${}_{3}^{+}$+F${}_{2}^{-}$→ C${}_{2}$F${}_{3}$+F${}_{2}$	1.0 $\times \;10^{-13}$	m${}^{3}$s${}^{-1}$	[34]
(R223)	CF${}_{2}^{+}$+F${}_{2}^{-}$→ CF${}_{2}$+F${}_{2}$	1.0 $\times \;10^{-13}$	m${}^{3}$s${}^{-1}$	[34]
(R224)	CF${}^{+}$+F${}_{2}^{-}$→ CF+F${}_{2}$	1.0 $\times \;10^{-13}$	m${}^{3}$s${}^{-1}$	[34]
(R225)	F${}_{2}^{+}$+F${}_{2}^{-}$→ 2F${}_{2}$	1.0 $\times \;10^{-13}$	m${}^{3}$s${}^{-1}$	[34]
(R226)	F${}^{+}$+F${}_{2}^{-}$→ F+F${}_{2}$	1.0 $\times \;10^{-13}$	m${}^{3}$s${}^{-1}$	[34]
(R227)	C${}^{+}$+F${}_{2}^{-}$→ C+F${}_{2}$	1.0 $\times \;10^{-13}$	m${}^{3}$s${}^{-1}$	[34]
(R228)	C${}_{4}$F${}_{7}^{+}$+F${}^{-}$→ C${}_{4}$F${}_{7}$+F	1.0 $\times \;10^{-13}$	m${}^{3}$s${}^{-1}$	[34]
(R229)	C${}_{3}$F${}_{7}^{+}$+F${}^{-}$→ C${}_{3}$F${}_{7}$+F	1.0 $\times \;10^{-13}$	m${}^{3}$s${}^{-1}$	[34]
(R230)	C${}_{3}$F${}_{6}^{+}$+F${}^{-}$→ C${}_{3}$F${}_{6}$+F	1.0 $\times \;10^{-13}$	m${}^{3}$s${}^{-1}$	[34]
(R231)	C${}_{3}$F${}_{5}^{+}$+F${}^{-}$→ C${}_{3}$F${}_{5}$+F	1.0 $\times \;10^{-13}$	m${}^{3}$s${}^{-1}$	[34]
(R232)	C${}_{2}$F${}_{5}^{+}$+F${}^{-}$→ C${}_{2}$F${}_{5}$+F	1.0 $\times \;10^{-13}$	m${}^{3}$s${}^{-1}$	[34]
(R233)	C${}_{2}$F${}_{4}^{+}$+F${}^{-}$→ C${}_{2}$F${}_{4}$+F	1.0 $\times \;10^{-13}$	m${}^{3}$s${}^{-1}$	[34]
(R234)	C${}_{2}$F${}_{3}^{+}$+F${}^{-}$→ C${}_{2}$F${}_{3}$+F	1.0 $\times \;10^{-13}$	m${}^{3}$s${}^{-1}$	[34]
(R235)	C${}_{4}$F${}_{7}^{+}$+F${}^{-}$→ C${}_{3}$F${}_{5}$+CF${}_{3}$	1.0 $\times \;10^{-13}$	m${}^{3}$s${}^{-1}$	[34]
(R236)	C${}_{3}$F${}_{7}^{+}$+F${}^{-}$→ C${}_{2}$F${}_{6}$+CF${}_{2}$	1.0 $\times \;10^{-13}$	m${}^{3}$s${}^{-1}$	[34]
(R237)	C${}_{3}$F${}_{6}^{+}$+F${}^{-}$→ C${}_{2}$F${}_{4}$+CF${}_{3}$	1.0 $\times \;10^{-13}$	m${}^{3}$s${}^{-1}$	[34]
(R238)	C${}_{3}$F${}_{5}^{+}$+F${}^{-}$→ C${}_{2}$F${}_{4}$+CF${}_{2}$	1.0 $\times \;10^{-13}$	m${}^{3}$s${}^{-1}$	[34]
(R239)	C${}_{2}$F${}_{4}^{+}$+F${}^{-}$→ CF${}_{2}$+CF+F${}_{2}$	1.0 $\times \;10^{-13}$	m${}^{3}$s${}^{-1}$	[34]
(R240)	CF${}_{3}^{+}$+F${}^{-}$→ CF${}_{2}$+2F	1.0 $\times \;10^{-13}$	m${}^{3}$s${}^{-1}$	[34]
(R241)	CF${}_{3}^{-}$+CF${}_{3}^{+}$→ 2CF${}_{3}$	1.0 $\times \;10^{-13}$	m${}^{3}$s${}^{-1}$	[33]
(R242)	CF${}_{3}^{-}$+CF${}_{2}^{+}$→ CF${}_{3}$+CF${}_{2}$	5.0 $\times \;10^{-13}$	m${}^{3}$s${}^{-1}$	[33]
(R243)	CF${}_{3}^{-}$+CF${}^{+}$→ CF${}_{3}$+CF	2.0 $\times \;10^{-13}$	m${}^{3}$s${}^{-1}$	[33]
(R244)	CF${}_{3}^{-}$+C${}^{+}$→ CF${}_{3}$+C	3.0 $\times \;10^{-13}$	m${}^{3}$s${}^{-1}$	[33]
(R245)	CF${}_{3}^{-}$+F${}_{2}^{+}$→ CF${}_{3}$+F${}_{2}$	2.0 $\times \;10^{-13}$	m${}^{3}$s${}^{-1}$	[33]
(R246)	CF${}_{3}^{-}$+F${}^{+}$→ CF${}_{3}$+F	2.5 $\times \;10^{-13}$	m${}^{3}$s${}^{-1}$	[33]
(R247)	F${}^{-}$+CF${}_{3}^{+}$→ CF${}_{4}$	5.0 $\times \;10^{-14}$	m${}^{3}$s${}^{-1}$	[33]
(R248)	F${}^{-}$+CF${}_{3}^{+}$→ CF${}_{3}$+F	1.0 $\times \;10^{-14}$	m${}^{3}$s${}^{-1}$	[33]
(R249)	F${}^{-}$+CF${}_{3}^{+}$→ CF${}_{2}$+F${}_{2}$	8.7 $\times \;10^{-14}$	m${}^{3}$s${}^{-1}$	[33]
(R250)	F${}^{-}$+CF${}_{2}^{+}$→ CF+F${}_{2}$	9.1 $\times \;10^{-14}$	m${}^{3}$s${}^{-1}$	[33]
(R251)	F${}^{-}$+CF${}_{2}^{+}$→ CF${}_{2}$+F	5.0 $\times \;10^{-13}$	m${}^{3}$s${}^{-1}$	[33]
(R252)	F${}^{-}$+CF${}^{+}$→ CF+F	4.0 $\times \;10^{-13}$	m${}^{3}$s${}^{-1}$	[33]
(R253)	F${}^{-}$+C${}^{+}$→ CF	1.2 $\times \;10^{-13}$	m${}^{3}$s${}^{-1}$	[33]
(R254)	F${}^{-}$+F${}_{2}^{+}$→ F+F${}_{2}$	9.4 $\times \;10^{-14}$	m${}^{3}$s${}^{-1}$	[33]
(R255)	F${}^{-}$+F${}^{+}$→ 2F	1.1 $\times \;10^{-13}$	m${}^{3}$s${}^{-1}$	[33]
(R256)	F${}_{2}^{-}$+CF${}_{3}^{+}$→ F${}_{2}$+CF${}_{3}$	1.0 $\times \;10^{-13}$	m${}^{3}$s${}^{-1}$	[33]

**Table A7:** C${}_{x}$F${}_{y}$ excitation reaction sets included in a volume-averaged plasma model.

	Reaction	Rate Constant	Unit	Reference
(R257)	e+CF${}_{2}$→ e+CF${}_{2}{(}^{3}B_{1})$	6.231 $\times \;10^{-14}$T${}_{e}^{-1.037}\exp\left(-3.964/T_{e}\right)$	m${}^{3}$s${}^{-1}$	[33]
(R258)	e+CF${}_{2}{(}^{3}B_{1})$→ e+CF${}_{2}{(}^{1}B_{1})$	3.528 $\times \;10^{-13}$T${}_{e}^{-0.877}\exp\left(-8.044/T_{e}\right)$	m${}^{3}$s${}^{-1}$	[33]
(R259)	e+CF${}_{2}$→ e+CF${}_{2}{(}^{1}B_{1})$	3.528 $\times \;10^{-13}$T${}_{e}^{-0.877}\exp\left(-10.254/T_{e}\right)$	m${}^{3}$s${}^{-1}$	[33]
(R260)	e+CF→ e+CF$\left(a^{4}\Sigma^{-}\right)$	6.132 $\times \;10^{-14}$T${}_{e}^{-1.173}\exp\left(-4.896/T_{e}\right)$	m${}^{3}$s${}^{-1}$	[33]
(R261)	e+F${}_{2}$→ e+F${}_{2}\left(v\right)$	2.586 $\times \;10^{-13}$T${}_{e}^{-1.446}\exp\left(-0.889/T_{e}\right)$	m${}^{3}$s${}^{-1}$	[33]
(R262)	e+F${}_{2}$→ e+F${}_{2}\left(C^{1}\Sigma_{u}\right)$	3.929 $\times \;10^{-15}$T${}_{e}^{0.665}\exp\left(-11.842/T_{e}\right)$	m${}^{3}$s${}^{-1}$	[33]
(R263)	e+F${}_{2}$→ e+F${}_{2}\left(H^{1}\Pi_{u}\right)$	9.616 $\times \;10^{-16}$T${}_{e}^{0.344}\exp\left(-17.274/T_{e}\right)$	m${}^{3}$s${}^{-1}$	[33]
(R264)	e+F→ e+F(3s ${}^{4}$P)	1.667 $\times \;10^{-15}$T${}_{e}^{-0.581}\exp\left(-12.851/T_{e}\right)$	m${}^{3}$s${}^{-1}$	[33]
(R265)	e+F→ e+F(3s ${}^{2}$P)	8.522 $\times \;10^{-16}$T${}_{e}^{-0.386}\exp\left(-12.351/T_{e}\right)$	m${}^{3}$s${}^{-1}$	[33]
(R266)	e+F→ e+F(3p ${}^{4}$P)	4.113 $\times \;10^{-16}$T${}_{e}^{-0.42}\exp\left(-14.261/T_{e}\right)$	m${}^{3}$s${}^{-1}$	[33]
(R267)	e+F→ e+F(3p ${}^{4}$D)	4.014 $\times \;10^{-16}$T${}_{e}^{-0.372}\exp\left(-14.629/T_{e}\right)$	m${}^{3}$s${}^{-1}$	[33]
(R268)	e+F→ e+F(3p ${}^{4}$D)	3.305 $\times \;10^{-16}$T${}_{e}^{-0.014}\exp\left(-14.062/T_{e}\right)$	m${}^{3}$s${}^{-1}$	[33]
(R269)	e+F→ e+F(3p ${}^{2}$S)	2.080 $\times \;10^{-16}$T${}_{e}^{-0.859}\exp\left(-15.269/T_{e}\right)$	m${}^{3}$s${}^{-1}$	[33]
(R270)	e+F→ e+F(3p ${}^{4}$S)	6.323 $\times \;10^{-17}$T${}_{e}^{-0.25}\exp\left(-14.703/T_{e}\right)$	m${}^{3}$s${}^{-1}$	[33]
(R271)	e+F→ e+F(3s ${}^{2}$P)	6.192 $\times \;10^{-16}$T${}_{e}^{-0.133}\exp\left(-14.31/T_{e}\right)$	m${}^{3}$s${}^{-1}$	[33]
(R272)	e+F→ e+F(3s ${}^{2}$D)	5.479 $\times \;10^{-16}$T${}_{e}^{-0.181}\exp\left(-14.558/T_{e}\right)$	m${}^{3}$s${}^{-1}$	[33]
(R273)	e+F→ e+F(4s ${}^{2}$D)	2.212 $\times \;10^{-16}$T${}_{e}^{-0.387}\exp\left(-15.625/T_{e}\right)$	m${}^{3}$s${}^{-1}$	[33]
(R274)	e+F→ e+F(4s ${}^{2}$P)	1.052 $\times \;10^{-16}$T${}_{e}^{0.42}\exp\left(-14.83/T_{e}\right)$	m${}^{3}$s${}^{-1}$	[33]
(R275)	e+F→ e+F(3d ${}^{4}$D)	7.268 $\times \;10^{-17}$T${}_{e}^{-0.475}\exp\left(-15.634/T_{e}\right)$	m${}^{3}$s${}^{-1}$	[33]
(R276)	e+F→ e+F(3d ${}^{2}$D)	5.733 $\times \;10^{-17}$T${}_{e}^{-0.474}\exp\left(-14.599/T_{e}\right)$	m${}^{3}$s${}^{-1}$	[33]
(R277)	e+F→ e+F(3d ${}^{4}$F)	7.340 $\times \;10^{-17}$T${}_{e}^{-0.556}\exp\left(-15.986/T_{e}\right)$	m${}^{3}$s${}^{-1}$	[33]
(R278)	e+F→ e+F(3d ${}^{2}$F)	7.253 $\times \;10^{-17}$T${}_{e}^{-0.252}\exp\left(-15.543/T_{e}\right)$	m${}^{3}$s${}^{-1}$	[33]
(R279)	e+F→ e+F(3d ${}^{4}$P)	4.668 $\times \;10^{-17}$T${}_{e}^{-0.819}\exp\left(-16.014/T_{e}\right)$	m${}^{3}$s${}^{-1}$	[33]
(R280)	e+F→ e+F(3d ${}^{2}$P)	2.008 $\times \;10^{-17}$T${}_{e}^{0.297}\exp\left(-14.749/T_{e}\right)$	m${}^{3}$s${}^{-1}$	[33]
(R281)	e+F→ e+F(4p ${}^{4}$P)	1.101 $\times \;10^{-16}$T${}_{e}^{-0.412}\exp\left(-16.132/T_{e}\right)$	m${}^{3}$s${}^{-1}$	[33]
(R282)	e+F→ e+F(4p ${}^{4}$D)	1.514 $\times \;10^{-16}$T${}_{e}^{-0.467}\exp\left(-16.207/T_{e}\right)$	m${}^{3}$s${}^{-1}$	[33]
(R283)	e+F→ e+F(4p ${}^{2}$D)	1.068 $\times \;10^{-16}$T${}_{e}^{-0.052}\exp\left(-15.789/T_{e}\right)$	m${}^{3}$s${}^{-1}$	[33]
(R284)	e+F→ e+F(4p ${}^{2}$S)	8.102 $\times \;10^{-17}$T${}_{e}^{-0.906}\exp\left(-16.669/T_{e}\right)$	m${}^{3}$s${}^{-1}$	[33]
(R285)	e+F→ e+F(4p ${}^{4}$S)	2.723 $\times \;10^{-17}$T${}_{e}^{-0.246}\exp\left(-16.014/T_{e}\right)$	m${}^{3}$s${}^{-1}$	[33]
(R286)	e+F→ e+F(4p ${}^{2}$P)	2.333 $\times \;10^{-16}$T${}_{e}^{-0.033}\exp\left(-15.86/T_{e}\right)$	m${}^{3}$s${}^{-1}$	[33]
(R287)	e+F→ e+F(5s ${}^{4}$P)	2.923 $\times \;10^{-16}$T${}_{e}^{-0.656}\exp\left(-17.789/T_{e}\right)$	m${}^{3}$s${}^{-1}$	[33]
(R288)	e+F→ e+F(5s ${}^{4}$P)	9.073 $\times \;10^{-17}$T${}_{e}^{0.249}\exp\left(-16.566/T_{e}\right)$	m${}^{3}$s${}^{-1}$	[33]
(R289)	e+C→ e+C(2s${}^{2}$2p${}^{2}$ ${}^{1}$D)	3.315 $\times \;10^{-14}$T${}_{e}^{-0.498}\exp\left(-1.995/T_{e}\right)$	m${}^{3}$s${}^{-1}$	[33]
(R290)	e+C→ e+C(2s${}^{2}$2p${}^{2}$ ${}^{1}$S)	4.900 $\times \;10^{-15}$T${}_{e}^{-0.584}\exp\left(-3.462/T_{e}\right)$	m${}^{3}$s${}^{-1}$	[33]
(R291)	e+C→ e+C(2s${}^{2}$2p${}^{3}$ ${}^{5}$S${}^{o}$)	3.831 $\times \;10^{-14}$T${}_{e}^{-0.813}\exp\left(-5.057/T_{e}\right)$	m${}^{3}$s${}^{-1}$	[33]
(R292)	e+C→ e+C(2s${}^{2}$2p${}^{3}$s ${}^{3}$P${}^{o}$)	4.751 $\times \;10^{-15}$T${}_{e}^{0.303}\exp\left(-6.984/T_{e}\right)$	m${}^{3}$s${}^{-1}$	[33]
(R293)	e+C→ e+C(2s${}^{2}$2p${}^{3}$s ${}^{1}$P${}^{o}$)	1.952 $\times \;10^{-15}$T${}_{e}^{-0.682}\exp\left(-8.019/T_{e}\right)$	m${}^{3}$s${}^{-1}$	[33]
(R294)	e+C→ e+C(2s${}^{2}$2p${}^{3}$ ${}^{3}$D${}^{o}$)	1.577 $\times \;10^{-14}$T${}_{e}^{0.036}\exp\left(-8.038/T_{e}\right)$	m${}^{3}$s${}^{-1}$	[33]
(R295)	e+C→ e+C(2s${}^{2}$2p${}^{3}$p ${}^{1}$P)	1.158 $\times \;10^{-15}$T${}_{e}^{-0.52}\exp\left(-9.008/T_{e}\right)$	m${}^{3}$s${}^{-1}$	[33]
(R296)	e+C→ e+C(2s${}^{2}$2p${}^{3}$p ${}^{3}$D)	3.160 $\times \;10^{-15}$T${}_{e}^{-0.176}\exp\left(-8.537/T_{e}\right)$	m${}^{3}$s${}^{-1}$	[33]
(R297)	e+C→ e+C(2s${}^{2}$2p${}^{3}$p ${}^{3}$S)	8.294 $\times \;10^{-16}$T${}_{e}^{-0.534}\exp\left(-8.762/T_{e}\right)$	m${}^{3}$s${}^{-1}$	[33]
(R298)	e+C→ e+C(2s${}^{2}$2p${}^{3}$p ${}^{3}$P)	3.872 $\times \;10^{-15}$T${}_{e}^{-0.026}\exp\left(-8.691/T_{e}\right)$	m${}^{3}$s${}^{-1}$	[33]
(R299)	e+C→ e+C(2s${}^{2}$2p${}^{3}$p ${}^{1}$D)	1.022 $\times \;10^{-15}$T${}_{e}^{-0.469}\exp\left(-9.391/T_{e}\right)$	m${}^{3}$s${}^{-1}$	[33]
(R300)	e+C→ e+C(2s${}^{2}$2p${}^{3}$p ${}^{1}$S)	1.043 $\times \;10^{-16}$T${}_{e}^{-0.45}\exp\left(-9.048/T_{e}\right)$	m${}^{3}$s${}^{-1}$	[33]
(R301)	e+C→ e+C(2s${}^{2}$2p${}^{3}$p ${}^{3}$P${}^{o}$)	8.132 $\times \;10^{-15}$T${}_{e}^{0.036}\exp\left(-9.942/T_{e}\right)$	m${}^{3}$s${}^{-1}$	[33]
(R302)	e+C→ e+C(2s${}^{2}$2p${}^{3}$p ${}^{1}$D${}^{o}$)	2.119 $\times \;10^{-16}$T${}_{e}^{-0.295}\exp\left(-9.836/T_{e}\right)$	m${}^{3}$s${}^{-1}$	[33]
(R303)	e+C→ e+C(2s${}^{2}$2p${}^{4}$s ${}^{3}$P${}^{o}$)	1.248 $\times \;10^{-15}$T${}_{e}^{-0.106}\exp\left(-9.574/T_{e}\right)$	m${}^{3}$s${}^{-1}$	[33]
(R304)	e+C→ e+C(2s${}^{2}$2p${}^{3}$d ${}^{3}$F${}^{o}$)	7.560 $\times \;10^{-16}$T${}_{e}^{-0.208}\exp\left(-9.620/T_{e}\right)$	m${}^{3}$s${}^{-1}$	[33]
(R305)	e+C→ e+C(2s${}^{2}$2p${}^{3}$d ${}^{3}$D${}^{o}$)	6.876 $\times \;10^{-16}$T${}_{e}^{0.578}\exp\left(-8.874/T_{e}\right)$	m${}^{3}$s${}^{-1}$	[33]
(R306)	e+C→ e+C(2s${}^{2}$2p${}^{4}$s ${}^{1}$P${}^{o}$)	3.275 $\times \;10^{-16}$T${}_{e}^{0.589}\exp\left(-9.982/T_{e}\right)$	m${}^{3}$s${}^{-1}$	[33]
(R307)	e+C→ e+C(2s${}^{2}$2p${}^{3}$d ${}^{1}$F${}^{o}$)	2.141 $\times \;10^{-16}$T${}_{e}^{-0.504}\exp\left(-10.083/T_{e}\right)$	m${}^{3}$s${}^{-1}$	[33]
(R308)	e+C→ e+C(2s${}^{2}$2p${}^{3}$d ${}^{1}$P${}^{o}$)	2.396 $\times \;10^{-16}$T${}_{e}^{-0.687}\exp\left(-9.973/T_{e}\right)$	m${}^{3}$s${}^{-1}$	[33]
(R309)	e+C→ e+C(2s${}^{2}$2p${}^{3}$ ${}^{1}$D)	1.315 $\times \;10^{-16}$T${}_{e}^{-0.594}\exp\left(-10.517/T_{e}\right)$	m${}^{3}$s${}^{-1}$	[33]
(R310)	e+C→ e+C(2s${}^{2}$2p${}^{3}$ ${}^{3}$S)	3.495 $\times \;10^{-16}$T${}_{e}^{-0.324}\exp\left(-10.193/T_{e}\right)$	m${}^{3}$s${}^{-1}$	[33]
(R311)	e+C→ e+C(2s${}^{2}$2p${}^{3}$ ${}^{1}$P)	7.857 $\times \;10^{-17}$T${}_{e}^{-0.458}\exp\left(-10.141/T_{e}\right)$	m${}^{3}$s${}^{-1}$	[33]
(R312)	e+C→ e+C(2s${}^{2}$2p${}^{3}$ ${}^{3}$P)	4.134 $\times \;10^{-16}$T${}_{e}^{-0.112}\exp\left(-10.168/T_{e}\right)$	m${}^{3}$s${}^{-1}$	[33]
(R313)	e+C→ e+C(2s${}^{2}$2p${}^{3}$ ${}^{1}$D)	1.388 $\times \;10^{-16}$T${}_{e}^{-0.567}\exp\left(-10.703/T_{e}\right)$	m${}^{3}$s${}^{-1}$	[33]
(R314)	e+C→ e+C(2s${}^{2}$2p${}^{3}$ ${}^{1}$S)	1.628 $\times \;10^{-17}$T${}_{e}^{-0.538}\exp\left(-10.681/T_{e}\right)$	m${}^{3}$s${}^{-1}$	[33]

**Table A8:** C${}_{x}$F${}_{y}$ charge exchange reaction set included in a volume-averaged plasma model.

	Reaction	Rate Constant	Unit	Reference
(R315)	C${}_{3}$F${}_{7}^{+}$+C${}_{2}$F${}_{4}$→ CF${}_{3}^{+}$+C${}_{4}$F${}_{8}$	2.0 $\times \;10^{-17}$	m${}^{3}$s${}^{-1}$	[34]
(R316)	C${}_{2}$F${}_{4}^{+}$+C${}_{2}$F${}_{4}$→ C${}_{3}$F${}_{5}^{+}$+CF${}_{3}$	2.0 $\times \;10^{-17}$	m${}^{3}$s${}^{-1}$	[34]
(R317)	CF${}_{3}^{+}$+C${}_{3}$F${}_{7}$→ C${}_{3}$F${}_{7}^{+}$+CF${}_{3}$	7.04 $\times \;10^{-16}$	m${}^{3}$s${}^{-1}$	[34]
(R318)	CF${}_{3}^{+}$+C${}_{3}$F${}_{5}$→ C${}_{3}$F${}_{5}^{+}$+CF${}_{3}$	7.04 $\times \;10^{-16}$	m${}^{3}$s${}^{-1}$	[34]
(R319)	CF${}_{3}^{+}$+C${}_{2}$F${}_{6}$→ C${}_{2}$F${}_{5}^{+}$+CF${}_{4}$	2.50 $\times \;10^{-18}$	m${}^{3}$s${}^{-1}$	[34]
(R320)	CF${}_{3}^{+}$+C${}_{2}$F${}_{4}$→ C${}_{3}$F${}_{7}^{+}$	3.30 $\times \;10^{-17}$	m${}^{3}$s${}^{-1}$	[34]
(R321)	CF${}_{2}^{+}$+C${}_{4}$F${}_{8}$→ C${}_{3}$F${}_{5}^{+}$+C${}_{2}$F${}_{4}$+F	2.10 $\times \;10^{-17}$	m${}^{3}$s${}^{-1}$	[34]
(R322)	CF${}_{2}^{+}$+C${}_{2}$F${}_{6}$→ C${}_{2}$F${}_{5}^{+}$+CF${}_{3}$	3.50 $\times \;10^{-17}$	m${}^{3}$s${}^{-1}$	[34]
(R323)	CF${}_{2}^{+}$+C${}_{2}$F${}_{4}$→ C${}_{2}$F${}_{4}^{+}$+CF${}_{2}$	1.00 $\times \;10^{-15}$	m${}^{3}$s${}^{-1}$	[34]
(R324)	CF${}^{+}$+C${}_{2}$F${}_{6}$→ CF${}_{3}^{+}$+C${}_{2}$F${}_{4}$	2.00 $\times \;10^{-16}$	m${}^{3}$s${}^{-1}$	[34]
(R325)	CF${}^{+}$+C${}_{2}$F${}_{4}$→ C${}_{3}$F${}_{5}^{+}$	1.30 $\times \;10^{-16}$	m${}^{3}$s${}^{-1}$	[34]
(R326)	CF${}^{+}$+C${}_{2}$F${}_{4}$→ CF${}_{3}^{+}$+2CF	2.60 $\times \;10^{-16}$	m${}^{3}$s${}^{-1}$	[34]
(R327)	F${}_{2}^{+}$+C${}_{2}$F${}_{5}$→ C${}_{2}$F${}_{5}^{+}$+F${}_{2}$	1.00 $\times \;10^{-16}$	m${}^{3}$s${}^{-1}$	[34]
(R328)	F${}_{2}^{+}$+C${}_{2}$F${}_{4}$→ C${}_{2}$F${}_{4}^{+}$+F${}_{2}$	1.00 $\times \;10^{-16}$	m${}^{3}$s${}^{-1}$	[34]
(R329)	F${}^{+}$+C${}_{2}$F${}_{6}$→ C${}_{2}$F${}_{5}^{+}$+F${}_{2}$	1.00 $\times \;10^{-15}$	m${}^{3}$s${}^{-1}$	[34]
(R330)	F${}^{+}$+C${}_{2}$F${}_{5}$→ C${}_{2}$F${}_{4}^{+}$+F${}_{2}$	1.00 $\times \;10^{-15}$	m${}^{3}$s${}^{-1}$	[34]
(R331)	F${}^{+}$+C${}_{2}$F${}_{4}$→ C${}_{2}$F${}_{3}^{+}$+F${}_{2}$	1.00 $\times \;10^{-15}$	m${}^{3}$s${}^{-1}$	[34]
(R332)	C${}_{4}$F${}_{8}^{-}$+F→ F${}^{-}$+C${}_{4}$F${}_{8}$	1.00 $\times \;10^{-15}$	m${}^{3}$s${}^{-1}$	[34]
(R333)	CF${}_{2}^{+}$+CF${}_{4}$→ CF${}_{3}^{+}$+CF${}_{3}$	4.00 $\times \;10^{-16}$	m${}^{3}$s${}^{-1}$	[33]
(R334)	CF${}_{2}^{+}$+CF${}_{3}$→ CF${}_{3}^{+}$+CF${}_{2}$	1.48 $\times \;10^{-15}$	m${}^{3}$s${}^{-1}$	[33]
(R335)	CF${}_{2}^{+}$+CF→ CF${}_{3}^{+}$+C	2.06 $\times \;10^{-15}$	m${}^{3}$s${}^{-1}$	[33]
(R336)	CF${}_{2}^{+}$+C→ CF${}^{+}$+CF	1.04 $\times \;10^{-15}$	m${}^{3}$s${}^{-1}$	[33]
(R337)	CF${}^{+}$+CF${}_{4}$→ CF${}_{3}^{+}$+CF${}_{2}$	1.80 $\times \;10^{-16}$	m${}^{3}$s${}^{-1}$	[33]
(R338)	CF${}^{+}$+CF${}_{3}$→ CF${}_{3}^{+}$+CF	1.71 $\times \;10^{-15}$	m${}^{3}$s${}^{-1}$	[33]
(R339)	CF${}^{+}$+CF${}_{2}$→ CF${}_{2}^{+}$+CF	1.00 $\times \;10^{-15}$	m${}^{3}$s${}^{-1}$	[33]
(R340)	C${}^{+}$+CF${}_{3}$→ CF${}_{3}^{+}$+C	5.00 $\times \;10^{-16}$	m${}^{3}$s${}^{-1}$	[33]
(R341)	C${}^{+}$+CF${}_{3}$→ CF${}_{2}^{+}$+CF	2.48 $\times \;10^{-15}$	m${}^{3}$s${}^{-1}$	[33]
(R342)	C${}^{+}$+CF${}_{2}$→ CF${}_{2}^{+}$+C	5.00 $\times \;10^{-16}$	m${}^{3}$s${}^{-1}$	[33]
(R343)	C${}^{+}$+CF→ CF${}^{+}$+C	3.18 $\times \;10^{-15}$	m${}^{3}$s${}^{-1}$	[33]
(R344)	F${}_{2}^{+}$+CF${}_{4}$→ CF${}_{3}^{+}$+F+F${}_{2}$	1.00 $\times \;10^{-16}$	m${}^{3}$s${}^{-1}$	[33]
(R345)	F${}_{2}^{+}$+CF${}_{3}$→ CF${}_{3}^{+}$+F${}_{2}$	1.00 $\times \;10^{-16}$	m${}^{3}$s${}^{-1}$	[33]
(R346)	F${}_{2}^{+}$+CF${}_{3}$→ CF${}_{3}^{+}$+2F	1.60 $\times \;10^{-15}$	m${}^{3}$s${}^{-1}$	[33]
(R347)	F${}_{2}^{+}$+CF${}_{2}$→ CF${}_{2}^{+}$+F${}_{2}$	1.00 $\times \;10^{-15}$	m${}^{3}$s${}^{-1}$	[33]
(R348)	F${}_{2}^{+}$+CF${}_{2}$→ CF${}_{3}^{+}$+F	1.79 $\times \;10^{-15}$	m${}^{3}$s${}^{-1}$	[33]
(R349)	F${}_{2}^{+}$+CF→ CF${}^{+}$+F	2.18 $\times \;10^{-15}$	m${}^{3}$s${}^{-1}$	[33]
(R350)	F${}_{2}^{+}$+C→ CF${}^{+}$+F	1.04 $\times \;10^{-15}$	m${}^{3}$s${}^{-1}$	[33]
(R351)	F${}_{2}^{+}$+CF${}_{4}$→ CF${}_{3}^{+}$+F${}_{2}$	1.00 $\times \;10^{-15}$	m${}^{3}$s${}^{-1}$	[33]
(R352)	F${}^{+}$+CF${}_{3}$→ CF${}_{3}^{+}$+F	1.00 $\times \;10^{-15}$	m${}^{3}$s${}^{-1}$	[33]
(R353)	F${}^{+}$+CF${}_{3}$→ CF${}_{2}^{+}$+F${}_{2}$	1.00 $\times \;10^{-15}$	m${}^{3}$s${}^{-1}$	[33]
(R354)	F${}^{+}$+CF${}_{2}$→ CF${}^{+}$+F${}_{2}$	2.28 $\times \;10^{-15}$	m${}^{3}$s${}^{-1}$	[33]
(R355)	F${}^{+}$+CF→ CF${}^{+}$+F	5.00 $\times \;10^{-16}$	m${}^{3}$s${}^{-1}$	[33]
(R356)	F${}^{+}$+CF→ C${}^{+}$+F${}_{2}$	2.71 $\times \;10^{-15}$	m${}^{3}$s${}^{-1}$	[33]
(R357)	F${}^{+}$+C→ C${}^{+}$+F	1.17 $\times \;10^{-15}$	m${}^{3}$s${}^{-1}$	[33]
(R358)	F${}^{+}$+F${}_{2}$→ F${}_{2}^{+}$+F	7.94 $\times \;10^{-16}$	m${}^{3}$s${}^{-1}$	[33]
(R359)	CF${}_{3}^{-}$+F→ CF${}_{3}$+F${}^{-}$	5.00 $\times \;10^{-14}$	m${}^{3}$s${}^{-1}$	[33]
